# Huotan Jiedu Tongluo Decoction Inhibits Balloon-Injury-Induced Carotid Artery Intimal Hyperplasia in the Rat through the PERK-eIF2*α*-ATF4 Pathway and Autophagy Mediation

**DOI:** 10.1155/2021/5536237

**Published:** 2021-07-20

**Authors:** Tenghui Tian, Keying Yu, Miao Zhang, Xiao Shao, Liping Chang, Rui Shi, Baojin Yao, Yue Deng

**Affiliations:** ^1^Changchun University of Chinese Medicine, Changchun 130117, Jilin, China; ^2^Hospital of Affiliated Changchun University of Chinese Medicine, Changchun 130021, Jilin, China; ^3^Jilin Ginseng Academy, Changchun University of Chinese Medicine, Changchun 130117, Jilin, China

## Abstract

In-stent restenosis (ISR) is the main factor affecting the outcome of percutaneous coronary intervention (PCI), and its main pathological feature is neointimal hyperplasia. Huotan Jiedu Tongluo decoction (HTJDTLD) is an effective traditional Chinese medicine (TCM) prescription for the treatment of vascular stenosis diseases. However, the precise anti-ISR mechanism of HTJDTLD remains unclear. Here, we investigated whether HTJDTLD can inhibit the excessive activation of endoplasmic reticulum stress (ERS) and reduce the level of autophagy factors through regulating the PERK-eIF2*α*-ATF4 pathway, thereby inhibiting the proliferation of the intima of blood vessels damaged by balloon injury (BI) and preventing the occurrence of ISR. In this study, a 2F Fogarty balloon was used to establish a common carotid artery (CCA) BI model in male Sprague-Dawley rats. Then, HTJDTLD (16.33 g/kg/d) or atorvastatin (1.19 mg/kg/d) was administered by gavage. Four weeks later, hematoxylin-eosin (HE) and Masson staining of the injured CCA were performed to observe the histological changes in the CCA. Immunohistochemistry (IHC) was used to assess the proliferation and dedifferentiation of vascular smooth muscle cells (VSMCs) in the CCA. Western blotting and RT-PCR were used to measure the expression of ERS- and autophagy-related proteins and mRNAs in the CCA. The results indicated that HTJDTLD significantly alleviated BI-induced carotid artery intimal hyperplasia and fibrosis and reduced the neointimal area (NIA) and NIA/medial area (MA) ratio. In addition, HTJDTLD inhibited the proliferation and dedifferentiation of VSMCs, reduced the expression of proliferating cell nuclear antigen (PCNA), and increased the smooth-muscle-*α*-actin- (SM*α*-actin-) positive area. HTJDTLD also significantly reduced the expression of the ERS-related factors: GRP78, p-PERK/PERK, p-eIF2*α*/eIF2*α*, ATF4, and CHOP. In addition, the expression of the autophagy-related factors, Beclin1, LC3B, and ATG12, was significantly decreased. In addition, in vitro experiments showed that HTJDTLD inhibited the above-mentioned ERS signal molecules in human umbilical vein endothelial cells (HUVEC) and rat aortic smooth muscle cells (A7R5) induced by tunicamycin (TM) and played a crucial role in protecting cells from damage. HTJDTLD may be a very promising drug for the treatment of ISR.

## 1. Introduction

Coronary heart disease (CHD) is an important disease that seriously threatens human life and health. In recent years, the morbidity and mortality of CHD have increased each year, and CHD has become the number one factor affecting human health [1]. Currently, percutaneous coronary intervention (PCI) is an important method for the treatment of CHD [2]. However, in-stent restenosis (ISR) caused by vascular remodeling after advanced PCI limits the long-term efficacy of this method [3]. High-resolution intracoronary imaging has shown that in the case of advanced stent failure after surgery, new atherosclerosis in the stent segment is the final common pathway of ISR events [4, 5]. Neointimal hyperplasia is considered to be the main cause of the development of new atherosclerosis leading to ISR after PCI [6].

Endoplasmic reticulum stress (ERS) is the response of various pathological factors to the accumulation of unfolded or misfolded proteins in the endoplasmic reticulum (ER), which interferes with ER homeostasis and leads to ER dysfunction [7]. The unfolded protein response (UPR), an evolutionarily conserved signaling cascade, is activated to protect the ER, reduce damage, and promote cell survival to thus maintain ER function and homeostasis [8]. However, when long-term ER stress cannot be resolved, it induces cell apoptosis [9]. Studies have shown that ERS can promote the formation of neointima and is considered to be an important factor in promoting atherosclerosis and ISR [10, 11]. Many studies have found that ERS can induce cell autophagy. Unfolded or misfolded proteins that accumulate in the ER are degraded by autophagy to reduce intracellular ERS. Research has indicated that ERS may induce autophagy through the protein kinase RNA-like endoplasmic reticulum kinase (PERK)-eukaryotic translation initiation factor 2*α* (eIF2*α* )-activating transcription factor 4 (ATF4) signaling pathway to reduce damage resulting from cell stress [12–14]. Under ERS conditions, glucose regulatory protein 78 (GRP78) triggers the autophagy cascade by activating the PERK pathway, which can directly or indirectly activate a variety of autophagy-related genes (such as Beclin1, LC3B, and ATG12) to reduce the effect of ERS on the vascular intima and prevent the occurrence of ISR [15, 16]. Therefore, basic autophagy is considered a protective response that can restore ER homeostasis [17]; however, in the case of severe damage, ERS excessively activates autophagy, which may cause autophagic death of cells, aggravate endothelial damage, and promote the development of ISR [18, 19]. The crosstalk between ERS and autophagy is complicated and needs to be further studied [20].

Although advancements in drug-eluting stents (DESs) in recent years have led to obvious improvements in the ability to alleviate ISR caused by neointimal hyperplasia [21], the restenosis rate is 5%–10% [22] and DESs are expensive. Treatments such as lipid-lowering drugs and dual antiplatelet therapy (DAPT) can also cause liver damage [23, 24] and high bleeding risk (HBR) [25]. It is necessary to identify safe and effective treatment methods, especially for patients who cannot tolerate DAPT [26]. Traditional Chinese medicine (TCM) prescriptions have shortcomings because they are often rough preparations, have unstable quality, and lack a clear mechanism of action. However, TCM formulations use natural ingredients, are relatively safe, have few side effects, and can treat diseases by affecting multiple targets with multiple ingredients; thus, they can be regarded as potential antiatherosclerotic drugs [27]. To date, TCM preparations have been widely used for the treatment of ISR and have achieved unique curative effects [28, 29].

Huotan Jiedu Tongluo decoction (HTJDTLD) is a TCM formula developed by Professor Yue Deng based on many years of clinical experience. It has the effects of eliminating phlegm, removing blood stasis, detoxification, and dredging collaterals. According to data from more than 20 years of clinical application, it can effectively treat vascular stenosis diseases. Previous domestic studies have found that HTJDTLD can treat ISR through exerting anti-inflammatory and antioxidative stress effects. However, the pathogenesis of ISR is complex, and the mechanism by which HTJDTLD exerts its antistenosis effect has not yet been elucidated.

The carotid artery balloon injury (BI) animal model has been widely used to study experimental restenosis [30]. In this study, a rat common carotid artery (CCA) BI model was established to evaluate the effect of HTJDTLD on the neointima after carotid artery BI in rats and identify its antirestenotic mechanism to explore whether the inhibition of ISR by HTJDTLD is related to the regulation of the PERK-eIF2*α*-ATF4 pathway and autophagy and to provide new pharmaceutical evidence for the efficacy of HTJDTLD in treating ISR after PCI.

## 2. Materials and Methods

### 2.1. Experimental Drug Preparation

The Chinese herbs that compose HTJDTLD, including Lonicerae Japonicae Flos (30 g), Radix Angelicae Sinensis (15 g), Radix et Rhizoma Nardostachyos (15 g), Radix et Rhizoma Salviae Miltiorrhizae (15 g), Radix Scrophulariae (15 g), Fructus Trichosanthis (20 g), Hirudo (5 g), Radix et Rhizoma Rhodiolae Crenulatae (15 g), and Radix et Rhizoma Glycyrrhizae (10 g), were provided by the Affiliated Hospital of Changchun University of Chinese Medicine (Chang Chun, China) and confirmed to be genuine by Deng Yue (Professor, Affiliated Hospital of Changchun University of Traditional Chinese Medicine, China). The medicinal materials were soaked for 30 minutes according to the protocol of the traditional water extraction method, the Chinese medicinal materials and water were decocted at a ratio of 1 : 8, and the mixture was cooked twice for 1 h each time, filtered, mixed, and subjected to rotary evaporation (IKA, Germany) to generate a working solution (1.633 g/ml). Atorvastatin calcium tablets (Pfizer, USA) were dissolved in purified water to prepare a working solution (0.119 mg/ml) and used as a positive control. The abovementioned drugs were stored at 4°C.

### 2.2. Analysis of HTJDTLD Components

High-performance liquid chromatography-tandem mass spectrometry (HPLC-MS/MS) was used to qualitatively analyze the main components of HTJDTLD. A Zorbax Eclipse C18 column (Agilent Technologies) was used, the column temperature was maintained at 30°C, the flow rate was 0.3 mL/min, the mobile phase consisted of 0.1% formic acid (A) and acetonitrile (B), the injection volume was 2 *μ*L, and the temperature of the injector was 4°C. The samples were analyzed in positive/negative mode under the following conditions: a temperature of 325°C, a sheath gas flow rate of 45 arb, an auxiliary gas flow rate of 15 arb, a purge gas flow rate of 1 arb, an electrospray voltage of 3.5 kV, a capillary temperature of 330°C, and a scan mode of first-level full scan (full scan, *m*/*z* 100–1500, resolution: 120,000) and second-level mass spectrometry scan (dd-MS2, Top*N* = 10, resolution: 60,000). The collision mode was set to high-energy collision dissociation (HCD). Finally, Compound Discoverer 3.1 was used to perform retention time correction, peak identification, peak extraction, etc. According to the information obtained from two-stage mass spectrometry, we identified and annotated the results (analyzed by China Qingdao Kechuang Quality Inspection Co., Ltd.).

### 2.3. Animals

Forty 5-week-old SPF male Sprague-Dawley rats weighing 380 ± 10 g were provided by Liaoning Changsheng Biotechnology Co., Ltd. (license number: SCXK 2015–0001) and housed in the SPF Barrier Animal Experimental Center of Changchun University of Traditional Chinese Medicine at a temperature of 20–26°C and a relative humidity of 40%–70% on a 12 h light-and-dark cycle. All experimental procedures were approved by the Institutional Animal Care and Use Committee of Changchun University of Chinese Medicine.

### 2.4. Model Establishment and Drug Treatment

The rats were anesthetized by intraperitoneal injection of 40 mg/kg 1% sodium pentobarbital (Sigma, USA) and fixed on an animal table. The neck skin was disinfected, and an incision was made along the median line of the neck. The left CCA was bluntly separated, the nerves were separated, the bifurcation of the internal and external carotid arteries was identified, the internal carotid artery and the proximal end of the CCA were clamped with a 0.5 cm microvascular clip, and the distal end of the external carotid artery was ligated with a 4–0 suture. The microscissors were used to cut a V-shaped opening in the external artery at an oblique angle of 45°, and the balloon catheter was inserted obliquely to a depth of approximately 3 cm with a guide wire, expanded with a pressure of 3.0–5.2 atm, and rotated back towards the branch of the vessel. This procedure was repeated 3 times. After recovery of blood flow and bleeding observation, the skin was sutured. Penicillin G (2 × 10^5^ U/d) was injected intramuscularly for 3 consecutive days after the operation to prevent infection. In the sham group, only the external artery was ligated.

The rats were randomly divided into 4 groups, namely, the sham group (Sham), BI group (BI), HTJDTLD group (BI + HTJDTLD), and atorvastatin group (BI + Atorvastatin), with 10 rats in each group. The HTJDTLD group was given 16.33 g/kg/d HTJDTLD by gavage, and the atorvastatin group was given 1.19 mg/kg/d atorvastatin by gavage. Similarly, the rats in the Sham group and BI group were given an equal volume of purified water. All rats were sacrificed under anesthesia after 12 h of fasting during the 4th weekend. A 3–4 cm piece of the damaged CCA was collected, the pathological samples were fixed with 4% paraformaldehyde, and the remaining samples were stored at −80°C for subsequent experiments.

### 2.5. Histological Assessment

Routine hematoxylin-eosin (HE) staining and Masson staining of the CCA were performed. The blood vessel specimens were fixed in 4% formaldehyde for 12–24 h, and 4 *μ*m thick paraffin-embedded tissue sections were cut. The slices were observed under a Leica optical microscope, and Image-Pro Plus 6.0 software (Media Cybernetics, USA) was used to measure the neointimal area (NIA) and medial area (MA) and calculate the NIA/MA ratio. Collagen fibers were stained blue with Masson's stain. The experiment was repeated 3 times for each group.

### 2.6. Immunohistochemistry (IHC)

Paraffin-embedded sections of the CCA were cut for each group of rats, routinely dewaxed, hydrated, and heated in citrate antigen retrieval solution for antigen retrieval. The sections were blocked with 5% bovine serum albumin (BSA) and then incubated with an antiproliferating cell nuclear antigen (PCNA) polyclonal antibody (1 : 200; Proteintech, China) and anti-SM*α*-actin polyclonal antibody (1 : 200; CST, USA) overnight at 4°C followed by biotin-labeled goat anti-rabbit IgG (1 : 2000; Abcam, USA) at 37°C in the dark for 30 min. Then, diaminobenzidine (DAB; MXB, China) was used for color development, images were captured and saved with the ISCapture system, and Image-Pro Plus 6 image processing software was used for data analysis.

### 2.7. Quantitative Real-Time RT-PCR

The qPCR method was used to further observe the effect of HTJDTLD on the ERS gene targets GRP78 and ATF4 and the autophagy gene targets Beclin1, LC3B, and ATG12 in the CCA of rats. Total RNA was extracted from the rat CCA using TRIzol reagent (Thermo, USA) according to the manufacturer's instructions and reverse transcribed into cDNA with PrimeScript RT Master Mix (Takara, JPN). Using the Bio-Rad CFX96 Touch system, qPCR was performed with TB Green Premix Ex Taq (Takara, JPN) in a reaction volume of 25 *μ*l by a two-step method with the cycle conditions: Stage 1: predenaturation, Repeat: 1, 95°C for 30 s; Stage 2: PCR reaction, Repeat: 40, 95°C for 5 s, 60°C for 30 s, and Stage 3 for dissociation (95°C for 10 s, 65°C for 30 s, and 95°C for 15 s). Gene expression was quantitatively analyzed by the 2^−ΔΔCT^ method. The threshold (Ct) value of the target gene was normalized to the expression of GAPDH. Primer 5.0 software was used to design the primers, and the target gene primers were synthesized by China Jilin Kumei Biotechnology Co., Ltd. The primers were as follows: GAPDH: F 5′-TACCCACGGCAAGTTCAA-3′ and R 5′-CGCTCCTGGAAGATGGTGAT-3′; GRP78: F 5′-CACTTGGTATTGAAACTGTGGG-3′ and R 5′-TGTTACGGTGGGCTGATTAT-3′; ATF4: F 5′-AGTCTGCCTTCTCCAGGTGTTC-3′ and R 5′-GCTGTCTTGTTTTGCTCCATCTT-3′; Beclin1: F 5′-GAGTCCCTGACAGACAAAT-3′ and R 5′-GAACAGTACAACGGCAAC-3′; LC3B: F 5′-ATAGAGCGATACAAGGGTG-3′ and R 5′-AGGAAGAAGGCTTGGTTA-3′; ATG12: F 5′-AAACGAAGAAATGGGCTGTG-3′ and R 5′-GAAGGGGCAAAGGACTGATT-3′; CHOP: F 5′-GAGAAGGAGCAGGAGAAC-3′ and R 5′-GACAGACAGGAGGTGATG-3′; and ATF6: F 5′-GATTGTGGGCGTCACTTCTCG-3′ and R 5′-TGGGATGCCAATGTTAGCCTG-3′.

### 2.8. Western Blot Analysis

Radio immunoprecipitation assay (RIPA) protein lysis buffer (Beyotime, China) was used to extract total protein from CCA tissue. A bicinchoninic acid (BCA) kit (Beyotime, China) was used to measure the protein concentration. An equal amount of each protein sample was separate by sodium dodecyl sulphate-polyacrylamide gel electrophoresis (SDS-PAGE; Solarbio, China) on a 10% separating gel and 5% stacking gel at 70 V for 30 min and then 140 V for 35 min. After electrophoresis, the proteins were transferred to polyvinylidene fluoride (PVDF; Roche, USA) membranes. The membranes were blocked with 5% skim milk for 1 h and then incubated with primary antibodies against glucose-regulated protein 78 (GRP78; 1 : 1000), phospho inositol-requiring enzyme 1 (IRE1p, Ser724; 1 : 1000), and eukaryotic translation initiation factor 2*α* (eIF2*α*; 1 : 2000) (Origene, USA); protein kinase R-like ER kinase (PERK; 1 : 1000), phospho-PERK (p-PERK, Thr980; 1 : 1000), phospho-eIF2*α* (p-eIF2*α*, Ser51; 1 : 1000), activating transcription factor 4 (ATF4; 1 : 1000), and CCAAT-enhancing binding protein homologous protein (CHOP; 1 : 1000) (CST, USA); p-PERK (Thr980; 1 : 1000; Thermo Fisher, USA); activating transcription factor 6 (ATF6, 1 : 1000) (Proteintech, China); autophagy related 12 (ATG12; 1 : 1000) and Beclin1 (1 : 500) (MyBioSource, USA); and glyceraldehyde-3-phosphate dehydrogenase (GAPDH; 1 : 2500) and microtubule-associated protein 1 light chain-3B (LC3B; 1 : 1000) (Abcam, USA) overnight at 4°C. Then, the membranes were incubated with a secondary antibody for 1 h and developed by enhanced chemiluminescence (ECL; Beyotime, China). Image-Pro Plus 6 software was used to analyze the relative level of each protein. GAPDH was used as an internal reference.

### 2.9. Effects of HTJDTLD on ERS in Vascular Endothelial Cells and Smooth Muscle Cells

HTJDTLD extract was freeze-dried by a Heto PowerDry LL3000 Freeze Dryer (Thermo, USA) and stored at −80°C; human umbilical vein endothelial cells (HUVEC, Shanghai Central Asia Institute of Biological Genetics, catalog number: #8000) and rat aortic smooth muscle cells (A7R5, Shanghai Zeye Biotechnology Co., Ltd., catalog number: ZY-R006) were cultured with high-glucose Dulbecco's modified eagle medium (DMEM, Gibco, USA) containing 10% fetal bovine serum in a cell incubator at 37°C and 5% CO_2_. The HUVEC and A7R5 cells in the logarithmic growth phase were seeded into a 96-well plate with 5 × 10^3^ cells and 4.5 × 10^3^ cells in each well, respectively. After 24 h, the supernatant was discarded and grouped according to the experiment (control group, TM (Sigma, USA) 0.25, 0.5, 1, 2, 4, 8, 16 *μ*g/mL groups). 100 *μ*L of the corresponding TM solution was added and cultured for 24 h. Then, 10 *μ*L of Cell Counting Kit-8 reagent (CCK-8 Kit, Shang bao, China) was added to each well and incubated with cells for about 2 h. The absorbance was measured at 450 nm using an Infinite 200 PRO plate reader (Life Sciences, USA). Cell viability was calculated as follows: cell viability (%) = (OD value of the experimental group-OD value of the blank group)/(OD value of the control group-OD value of the blank group) × 100%. Same methods as above were used to investigate the effects of HTJDTLD on TM-induced cell damage on HUVEC and A7R5 cells, including control group, TM induced HUVEC group, or TM induced A7R5 group with HTJDTLD at concentrations of 0, 0.25, 0.5, 0.1, 0.2, 0.4, 0.8, 1.6, 3.2 mg/mL. According to the optimal doses obtained from the CCK-8 assay, the protein extracts were prepared from control group, TM-induced group (HUVEC; A7R5), HTJDTLD-treated group, and Atorvastatin-treated group (positive drug control) by seeding the cells into 6-well plates and being cultured for 24 h. Western blotting was performed to detect ERS-related signal molecules.

### 2.10. Statistical Analysis

All data are expressed as the mean ± S.D. and were analyzed by one-way ANOVA using Stata 25.0 (SPSS, Inc., Chicago, IL). Tukey's multiple comparison test was used to analyze differences between groups. *P* < 0.05 was considered statistically significant.

## 3. Results

### 3.1. Quality Control of HTJDTLD

**Figure 1:**
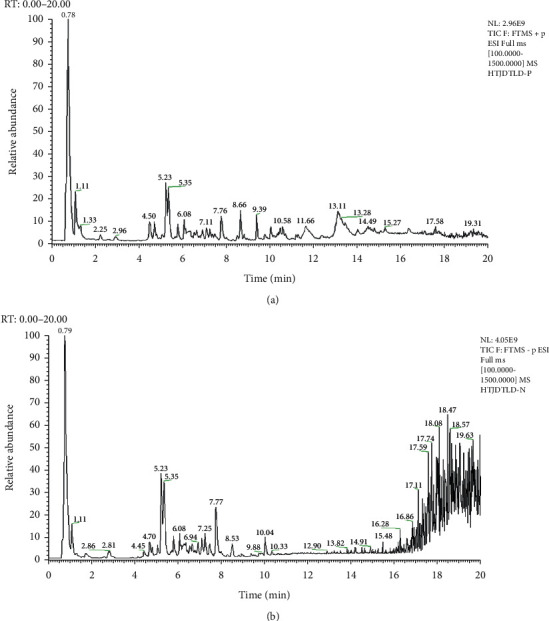
The TIC diagram of HTJDTLD. (a) Positive ion mode. (b) Negative ion mode.

HPLC-MS/MS was used to analyze and identify the main components of HTJDTLD. TIC diagrams were obtained in positive ion mode and negative ion mode (Figures 1(a) and 1(b)). We found that the main peak was more obvious in positive ion mode than in negative ion mode. The six most abundant chemical components (in positive ion mode) are listed in Table 1. The most abundant components were organooxygen compounds, carboxylic acids and its derivatives, and isobenzofurans. The results of the full-spectrum analysis are shown in the Supplementary Material file, which also contains all the specific ingredients and their classifications. Through identification, chlorogenic acid, salvianolic acid A, ferulic acid, salvianolic acid B, harpagide, tanshinone IIA, dihydrotanshinone I, and nardosinone licorice saponin G2 were confirmed to be present in HTJDTLD.

### 3.2. HTJDTLD Inhibits Neointimal Formation and Fibrosis after Carotid Artery BI in Rats

**Figure 2:**
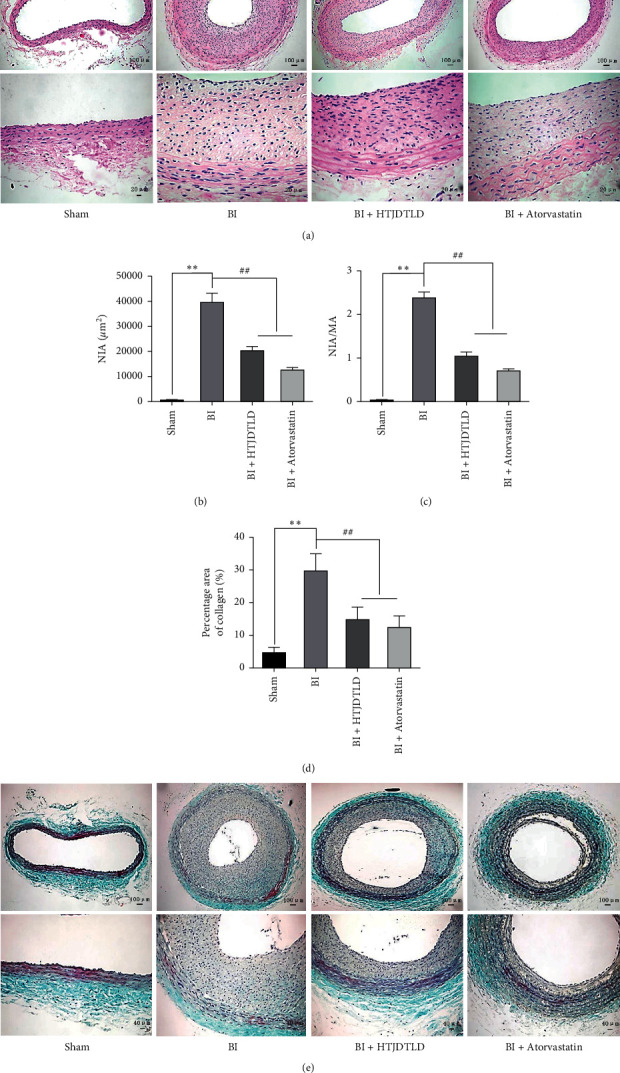
The effect of HTJDTLD on neointima formation and fibrosis of the CCA after BI in rats. (a) HE staining was performed to assess the effect of HTJDTLD on neointima in the CCA (original magnification: 100× or 400×). (b) NIA of the CCA for rats in each group. (c) Average NIA/MA ratio. (d) Percentage of fibrosis area in the rat carotid artery. (e) Masson staining of the CCAs of rats in each group was performed to evaluate the effect of HTJDTLD on deposition and fibrosis (original magnification: 100× or 200×) (*n* = 10, mean ± SD). ^*∗∗*^*P* < 0.01 vs. the Sham group, ^##^*P* < 0.01 vs. the BI group.

HE staining showed that the carotid artery was significantly narrower and that the intima was thicker in the BI group than in the Sham group (*P* < 0.01). However, HTJDTLD alleviated intimal hyperplasia caused by BI and increased the lumen area (Figure 2(a)). The results showed that the NIA and NIA/MA ratios were significantly higher in the BI group than the Sham group (*P* < 0.01), while the NIA and NIA/MA ratios were significantly lower in the HTJDTLD group and the atorvastatin group than that in the BI group (*P* < 0.01) (Figures 2(b) and 2(c)).

In addition, Masson staining showed that carotid artery collagen deposition and the percentage of fibrosis area in the BI group were significantly higher than those in the Sham group (*P* < 0.05); compared with BI, HTJDTLD and atorvastatin reduced carotid artery collagen deposition and the percentage of fibrosis area (*P* < 0.05) (Figures 2(d) and 2(e)).

### 3.3. HTJDTLD Can Regulate the Expression of PCNA and SM*α*-Actin in the Neointima of the CCA after BI in Rats

**Figure 3:**
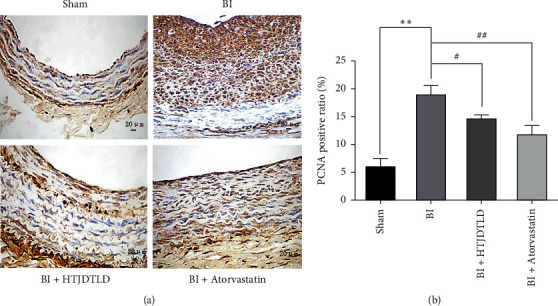
The effect of HTJDTLD on the percentage of PCNA-positive area in the CCA following BI. (a) Representative images of immunohistochemical staining for PCNA in sections of the CCA from each group (original magnification: 400×). (b) Percentage of PCNA-positive area in each group (*n* = 10, mean ± SD). ^*∗∗*^*P* < 0.01 vs. the Sham group. ^#^*P* < 0.05, ^##^*P* < 0.01 vs. the BI group.

Twenty-eight days after BI, immunohistochemistry was performed to assess the effect of HTJDTLD on the proliferation of vascular smooth muscle cells (VSMCs) in the CCA intima. PCNA-positive cells were stained brownish yellow. There was almost no PCNA-positive brown cells in the inner membrane in the Sham group, while the intima of the BI group had a large number of brown PCNA-positive cells (Figure 3(a)). Importantly, the data showed that, compared with BI, HTJDTLD and atorvastatin significantly reduced the percentage of the PCNA-positive area in the vessel wall following BI (*P* < 0.05) (Figure 3(b)).

**Figure 4:**
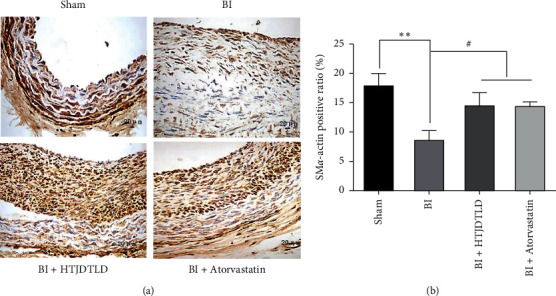
The effect of HTJDTLD on the percentage of SM*α*-actin-positive area in the CCA of rats following BI. (a) Representative images of immunohistochemical staining for SM*α*-actin in slices of CCA from each group (original magnification: 400 ×). (b) Average density of SM*α*-actin-positive cells in the carotid artery intima in each group (*n* = 10, mean ± SD). ^*∗∗*^*P* < 0.01 vs. the Sham group; ^#^*P* < 0.05 vs. the BI group.

In addition, we performed immunohistochemistry to evaluate the effect of HTJDTLD on the phenotypic regulation of VSMCs in the inner membrane following BI (Figure 4). The results revealed that SM*α*-actin staining was obvious in the CCA in the Sham group, significantly weakened in the BI group, and significantly increased in the CCAs of rats in the HTJDTLD and atorvastatin groups. The results showed that the positive staining area of the arterial intima in the Sham group was larger than that in the BI group (*P* < 0.01). Compared with BI, HTJDTLD or atorvastatin treatment significantly increased the area of SM*α*-actin staining (*P* < 0.05).

### 3.4. Effect of HTJDTLD on ERS in the CCA in Rats after BI

**Figure 5:**
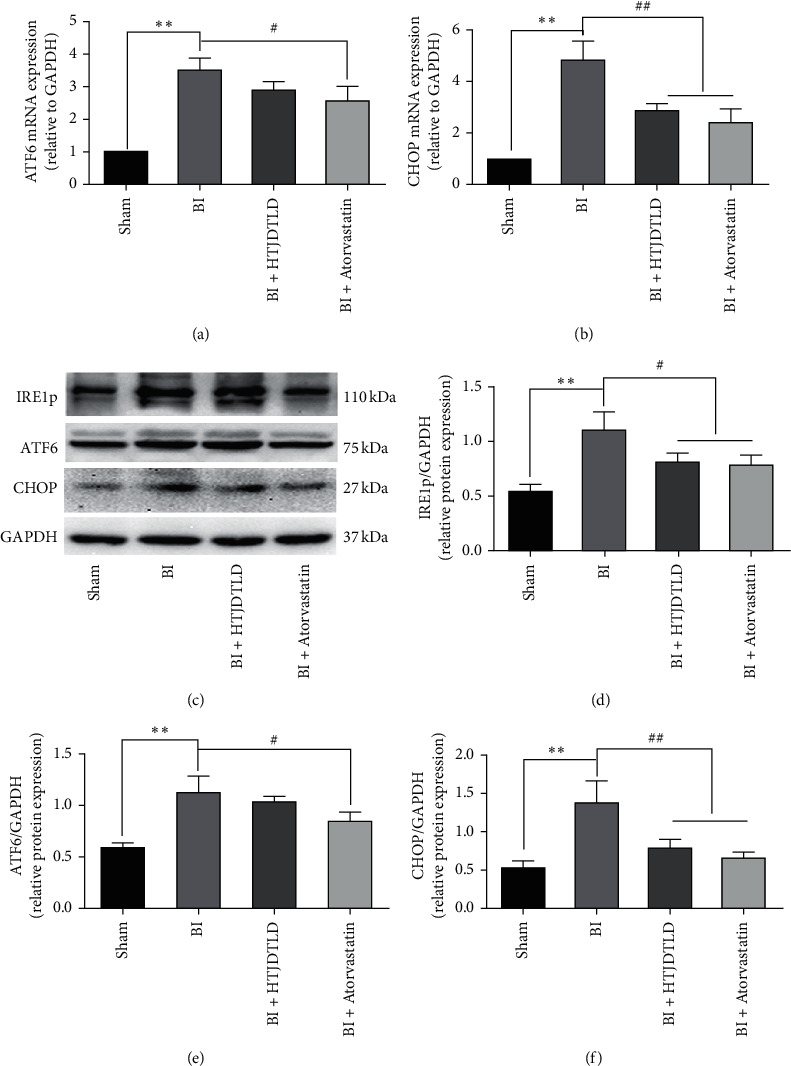
The effect of HTJDTLD on the expression of IRE1p, ATF6, and CHOP (*n* = 10, mean ± SD). ^*∗∗*^*P* < 0.01 vs. the Sham group. ^#^*P* < 0.05, ^##^*P* < 0.01 vs. the BI group.

**Figure 6:**
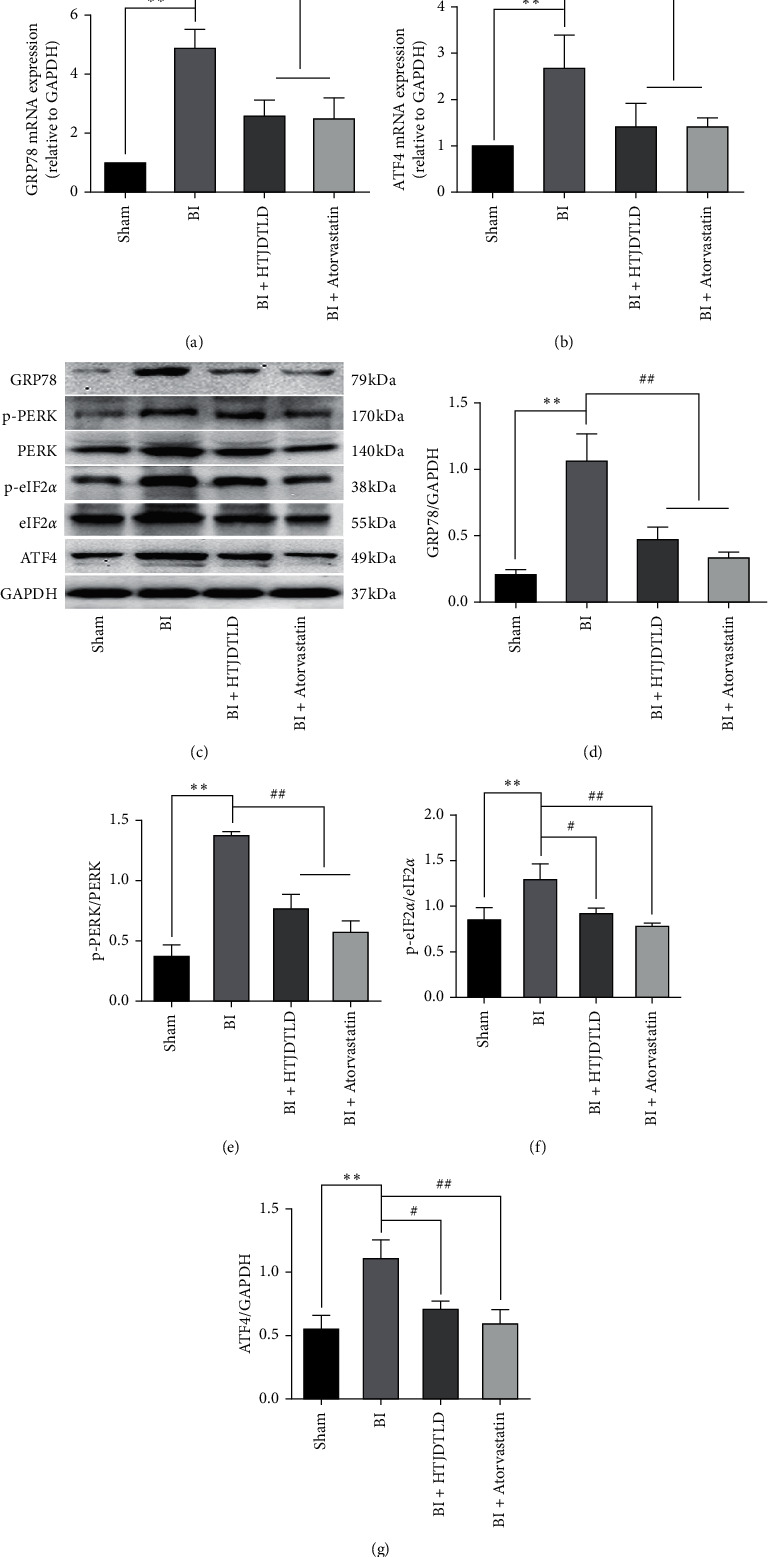
The effect of HTJDTLD on the expression of GRP78, p-PERK/PERK, p-eIF2*α*/eIF2*α,* and ATF4 (*n* = 10, mean ± SD). ^*∗∗*^*P* < 0.01 vs. the Sham group. ^#^*P* < 0.05, ^##^*P* < 0.01 vs. the BI group.

The results showed that the expression of GRP78 in the BI group was significantly higher than that in the Sham group (*P* < 0.01), indicating that BI activated the ERS response in the CCA. We further observed the initial signals of the three stress pathways of the UPR: PERK, IRE1, and ATF6. We found that BI increased the expression of PERK, IRE1p, and ATF6 in the CCA (*P* < 0.05). However, the effect of HTJDTLD on ATF6 was not obvious (*P* > 0.05) (Figure 5), and the inhibitory effect of HTJDTLD on PERK was more significant than its inhibitory effect on IRE1p (*P* < 0.01). Therefore, we further studied the changes in the PERK pathway and its downstream signals. The data showed that compared with that in the Sham group, the protein expression of GRP78, p-PERK/PERK, p-eIF2*α*/eIF2*α*, ATF4, and CHOP in the BI group was significantly increased (*P* < 0.05). In addition, the mRNA expression of GRP78 and ATF4 was significantly increased (*P* < 0.05). HTJDTLD and atorvastatin significantly inhibited these phenomena (Figure 6). These data indicate that HTJDTLD can effectively inhibit the PERK-eIF2*α*-ATF4 pathway.

### 3.5. HTJDTLD Inhibits the Expression of the Autophagy-Related Factors Beclin1, LC3B, and ATG12 in the CCA in Rats after BI

**Figure 7:**
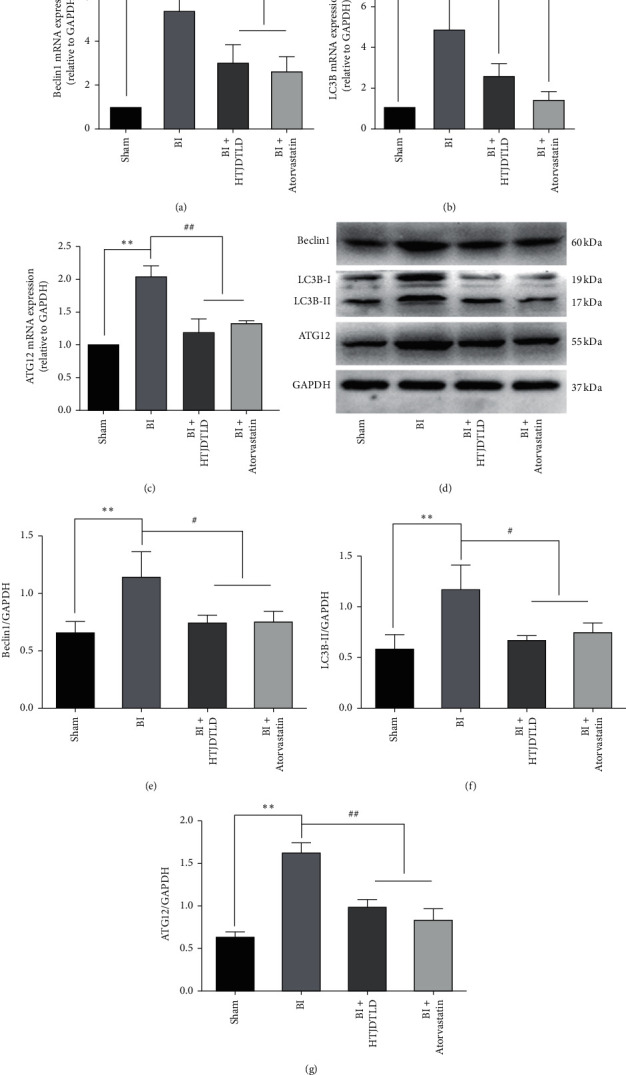
The effect of HTJDTLD on the expression of Beclin1, LC3B, and ATG12. (a)‐(c) RT-PCR analysis of the mRNA expression of Beclin1, LC3B, and ATG12. (d)‐(g) Western blot analysis of Beclin1, LC3B, and ATG12 protein expression in the CCA in each group (*n* = 10, mean ± SD). ^*∗*^*P* < 0.05, ^*∗∗*^*P* < 0.01 vs. the Sham group. ^#^*P* < 0.05, ^##^*P* < 0.01 vs. the BI group.

RT-PCR showed that the mRNA expression levels of Beclin1, LC3B, and ATG12 in the BI group were significantly higher than those in the Sham group (*P* < 0.05). It is worth noting that HTJDTLD and atorvastatin significantly attenuated the expression of these autophagy-related factors (*P* < 0.05) (Figures 7(a)–7(c)), and similar results were obtained by western blot analysis (Figures 7(d)–7(g)).

### 3.6. The Effects of HTJDTLD on HUVEC and A7R5 Cell under the Treatment of TM-Induced Injury

**Figure 8:**
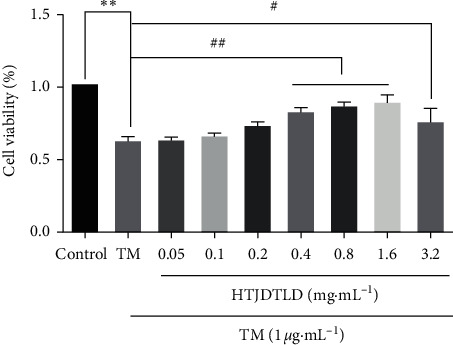
The effects of HTJDTLD on HUVEC cell viability under the treatment of TM-induced injury (*n* = 3, mean ± SD). ^*∗∗*^*P* < 0.01 vs. the Control group; ^##^*P* < 0.01, ^#^*P* < 0.05 vs. the TM group.

Compared with the TM group [31], cell viabilities under the treatment of HTJDTLD were increased with a dose-dependent manner and reached a maximum activity at 1.6 mg/mL, as shown in Figure 8. Thus, this concentration was selected for the subsequent experiments.

**Figure 9:**
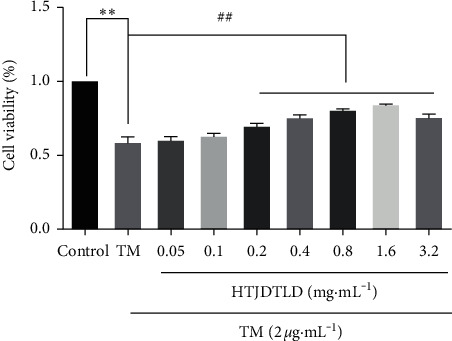
The effects of HTJDTLD on A7R5 cell viability under the treatment of TM-induced injury (*n* = 3, mean ± SD). ^*∗∗*^*P* < 0.01 vs. the Control group; ^##^*P* < 0.01 vs. the TM group.

Similar results were obtained from the A7R5 cells. Compared with the TM group [32], cell viabilities under the treatment of HTJDTLD were increased with a dose-dependent manner and reached a maximum activity at 1.6 mg/mL, as shown in Figure 9. Thus, this concentration was selected for the subsequent experiments.

**Figure 10:**
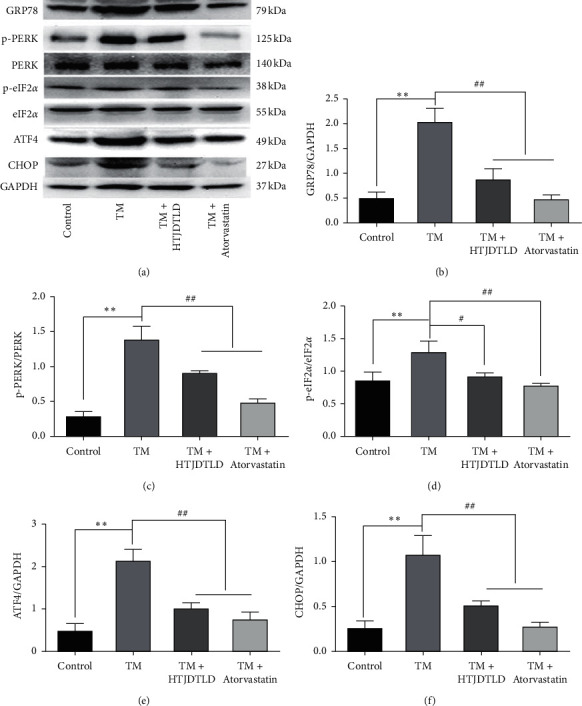
Effects of HTJDTLD on ERS signal molecules after TM-induced injury of HUVEC cells (*n* = 3, mean ± SD). ^*∗∗*^*P* < 0.01 vs. the control group; ^##^*P* < 0.01, ^#^*P* < 0.05 vs. the TM group. TM group (TM 1 *μ*g/mL), HTJDTLD group (TM 1 *μ*g/mL + HTJDTLD 1.6 mg/mL), and Atorvastatin group (TM 1 *μ*g/mL + Atorvastatin 10 *μ*M) [33].

**Figure 11:**
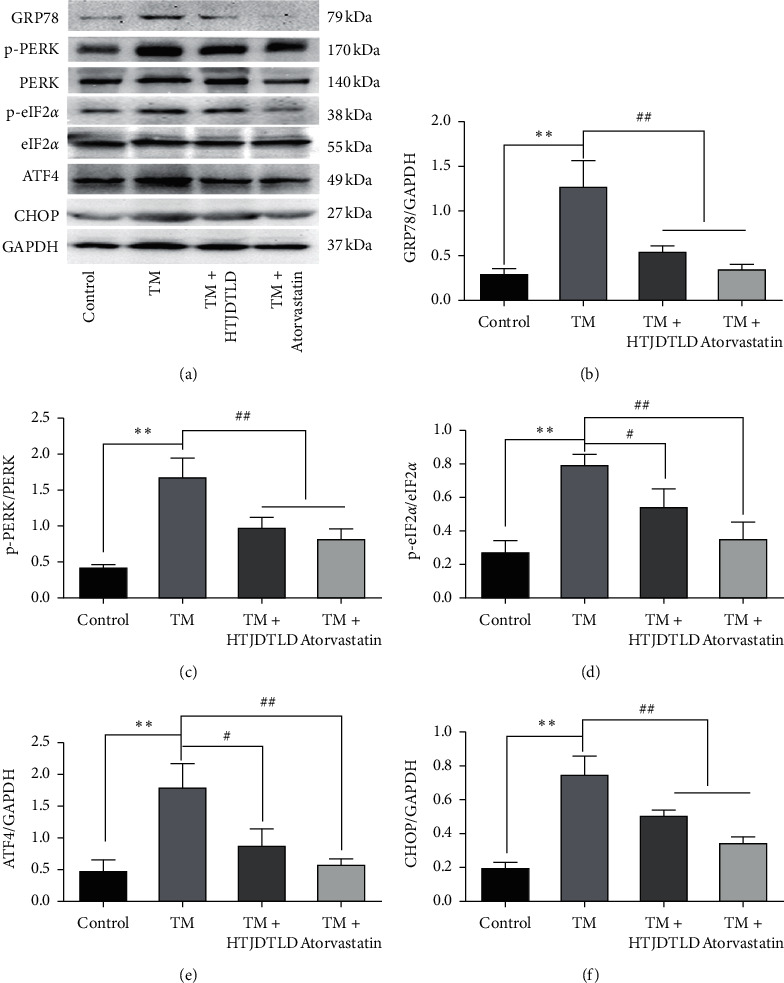
Effects of HTJDTLD on ERS signal molecules after TM-induced injury of A7R5 cells (*n* = 3, mean ± SD). ^*∗∗*^*P* < 0.01 vs. the Control group; ^##^*P* < 0.01, ^#^*P* < 0.05 vs. the TM group. TM group (TM 2 *μ*g/mL), HTJDTLD group (TM 2 *μ*g/mL + HTJDTLD 1.6 mg/mL), and Atorvastatin group (TM 2 *μ*g/mL + Atorvastatin 10 *μ*M).

Western blot results showed that the protein expression levels of ERS-related molecules including GRP78, p-PERK/PERK, p-eIF2*α*/eIF2*α*, ATF4, and CHOP were increased in the TM group (*P* < 0.05), but decreased in the HTJDTLD and Atorvastatin-treated groups, as shown in Figure 10 (HUVEC), Figure 11 (A7R5).

## 4. Discussion

ISR caused by neointimal hyperplasia is a concern after PCI treatment. As DESs and oral drug treatments are associated with the risk of ISR and side effects, Chinese medicine formulations may have the unique potential to alleviate ISR and reduce the side effects of treatment. HTJDTLD is a Chinese medicine prescription that is used for the clinical treatment of vascular stenosis diseases. In this study, we established a rat model of CCA BI and, for the first time, confirmed that HTJDTLD reduces the NIA and NIA/MA ratio in the rat CCA following BI and decreases the content of collagen fibers in the CCA. The results showed that HTJDTLD can inhibit CCA neointima formation and fibrosis after carotid artery BI in rats and prevent the ISR.

VSMCs proliferation is the main contributor to intimal hyperplasia after PCI [34]. When the endothelium is damaged, VSMCs proliferate and migrate to the new inner membrane space while secreting a large amount of matrix protein [35]. Therefore, inhibiting blood VSMCs proliferation and migration may be an effective method for preventing ISR after PCI [36]. PCNA is an important factor in DNA replication and repair, and the PCNA protein level is increased in cells in S phase and decreased in quiescent or senescent cells [37]. It is a marker of VSMCs proliferation. This study showed that the area of PCNA-positive cells in the CCA in the BI group was significantly increased and that HTJDTLD treatment decreased the area of PCNA-positive cells, indicating that HTJDTLD inhibits VSMCs proliferation. In addition, when VSMCs proliferate and migrate, they transition from the highly differentiated nonproliferative phenotype (contractile phenotype) to the poorly differentiated proliferative phenotype (synthetic phenotype) [38], which is key to promoting VSMCs proliferation. SM*α*-actin is a marker of VSMCs differentiation. Downregulation of SM*α*-actin expression is considered to be an important indicator of VSMCs differentiation. The expression of SM*α*-actin is negatively correlated with PCNA expression [36]. IHC showed that, compared with that in the Sham group, the percentage of SM*α*-actin-positive area in the BI group was decreased. After HTJDTLD treatment, the percentage of SM*α*-actin-positive area increased. This finding may suggest that HTJDTLD exerts a protective effect against ISR by inhibiting the proliferation and phenotypic transition of VSMCs in the CCA following BI, which is consistent with the results of histomorphological. Previous studies have shown that ERS plays a key role in intimal hyperplasia caused by the proliferation of VSMCs after vascular injury and that inhibition of ERS can prevent intimal hyperplasia [39]. However, whether the inhibitory effect of HTJDTLD on intimal hyperplasia in the CCA after BI is achieved by inhibiting ERS is not yet known. Therefore, we further explored the potential mechanism by which HTJDTLD protects against stenosis.

The effect of ERS on ISR after PCI is a relatively new research focus. Inhibiting ERS may be a new method for treating ISR [40]. It is known that PERK, IRE1, and ATF6, the three main stress sensors, which are located on the ER membrane, can activate the UPR [41]. GRP78 usually binds to these three key transmembrane protein receptors when they are inactive. When ERS is induced, GRP78 dissociates from the receptors, thereby initiating the UPR signaling cascade. It is worth noting that although previous studies have reported that the IRE1 or ATF6 pathway plays a role in ISR and other vascular diseases [42, 43], this study showed that ATF6 is not sensitive to the effect of HTJDTLD and that the inhibitory effect of HTJDTLD on PERK is stronger than its inhibitory effect on IRE1. The latest research has shown that inhibiting the PERK pathway can inhibit the development of ISR and thus may be used as a new treatment strategy for ISR [44]. In the PERK pathway, PERK acts as a sensor for the UPR, and after dissociating from GRP78, it activates eIF2*α* by phosphorylating it and simultaneously activates ATF4 in a context-dependent manner. The PERK pathway can reduce protein synthesis and slow protein translation, which can alleviate ERS [20]. However, excessive or persistent activation of the UPR can induce the activation of apoptotic factors through ATF4-CHOP signaling in the PERK pathway [45]. This activation of apoptotic factors has been confirmed to be an important contributor to many cardiovascular diseases [46]. The results of this study show that BI can increase the expression of GRP78, ATF4, p-PERK, and p-eIF2*α* in the CCA, indicating that BI activates the PERK pathway in the CCA. In addition, the expression of the ERS-related proapoptotic factor CHOP is significantly increased, indicating that severe BI may induce cell apoptosis through the PERK pathway and aggravate vascular endothelial damage. It is worth noting that the expression of these factors was significantly reduced after HTJDTLD treatment. This finding combined with histomorphological analysis of the CCA suggests that HTJDTLD inhibits ERS-induced vascular endothelial damage, thereby alleviating intimal hyperplasia. The protective effect of HTJDTLD may be related to the inhibition of the PERK-eIF2*α*-ATF4 signaling pathway.

ERS-induced autophagy can cooperatively reduce ERS and have an antivascular stenosis effect [47]. According to reports, ERS-induced autophagy does not require ATF6, but the PERK-eIF2*α*-ATF4 pathway is very important for this process [48]. The PERK-eIF2*α*-ATF4 signaling pathway has been proven to be an essential factor in inducing Atg12 expression and LC3 conversion, and it is a key mediator of ERS-induced autophagy [15]. ATF4 can transcriptionally regulate the expression of Atg12, induce the formation of the Atg5-Atg12-Atg16L complex, and regulate autophagy elongation [49]. Beclin1 is thought to be highly related to PERK in the UPR pathway and is regulated by ATF4, which depends on PERK activation. The PERK-ATF4-Beclin1 pathway has been shown to play an important role in ERS-related autophagy [50]. The results of this study showed that the protein and mRNA levels of the autophagy-related factors, Beclin1, LC3B, and ATG12, were increased in the CCA after BI, showing that BI injury activates autophagy in the CCA, reduces endothelial damage through autophagy, and promotes cell survival. It is worth noting that HTJDTLD treatment reduced the expression of these autophagy-related factors and reduced the autophagy response, possibly by inhibiting the excessive activation of factors upstream of the ERS pathway and reducing ERS-induced damage. However, some studies have shown that, under special circumstances, overactivation of autophagy can also promote cell apoptosis [20], which may aggravate endothelial damage and promote the development of ISR. At present, the interaction between ERS and autophagy and the duality of autophagy are relatively complicated, and future in-depth scientific research is needed. However, regardless of whether ERS-induced autophagy plays a role in alleviating ERS to achieve protection or inducing cell death and damage, HTJDTLD alleviated the endothelial damage caused by ERS in the CCA and then reduced the activation of autophagy to inhibit the development of ISR in this experiment.

Atorvastatin is currently the first-line drug in the clinic after PCI [51]. In addition to lowering cholesterol, atorvastatin can exert anti-inflammatory and antioxidant effects and inhibit VSMCs proliferation [52–54]. Studies have shown that atorvastatin can inhibit the formation of new intima in the rat CCA caused by BI [55], and there have also been reports that atorvastatin can inhibit ERS [56]. Therefore, atorvastatin was used as the positive control in this experiment. According to the experimental results, both HTJDTLD and atorvastatin have a significant inhibitory effect on neointimal hyperplasia in the rat CCA and have similar effects on the ERS and autophagy signaling pathways. However, research has revealed that atorvastatin is hepatotoxic [57] and can cause statin-induced myopathy (SIM) [58]. However, as HTJDTLD has been shown to have relatively few side effects and a stable efficacy over many years of clinical administration, it can be considered an alternative strategy for the treatment of ISR.

In addition, according to the in vitro experiments, the results showed that TM (a typical agent that can induce ERS by interfering with the glycosylation of N-linked protein in ER) can cause ERS stress in both endothelial cells and smooth muscle cells, and excessive ERS can induce cell damage and apoptosis, which suppress cell viability [59]. In our study, HTJDTLD can inhibit ERS in both the two kinds of cells including HUVEC and A7R5 and reduce cell damage under ERS condition, subsequently promoting cell survival and damage repair. As we know, endothelial cells are the main component of vascular intima, and the implementation of balloon injury can directly lead to the damage or exfoliation of the intima and then cause a strong ERS response to affect the intima and media. Thus, the smooth muscle cells suffered a strong ERS-related damage and migrated to the neointima, resulting in the thickening of the intima and the formation of stenosis [60]. Therefore, we speculate that HTJDTLD might promote the repair of endothelial cells and reduce the migration ability of smooth muscle cells induced by ERS to achieve the purpose of inhibiting the formation of new intima. On the other hand, combined with the results of in vivo experiments, we speculate that HTJDTLD might have better endothelial repair ability compared to smooth muscle cells and accelerate the reendothelialization after vascular injury [61]. Thus, when the endothelial cell damage is repaired in time, the ERS in the damaged blood vessel is reduced and the healed endothelium forms a barrier, which further inhibits the transmission of ERS to the smooth muscle cells in the media, thereby inhibiting the formation of new intima, subsequently preventing vascular stenosis.

## 5. Conclusions

**Figure 12:**
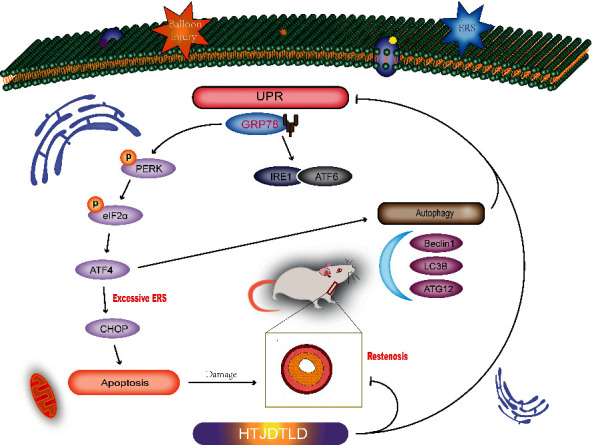
The regulatory mechanism of HTJDTLD in the rat model of CCA BI.

In summary, HTJDTLD effectively reduces proliferation and fibrosis in the neointima in rat CCA following BI and inhibits VSMCs proliferation and phenotypic conversion. In addition, HTJDTLD inhibits the PERK-eIF2*α*-ATF4 pathway and reduces the activation of the downstream autophagy pathway, thereby playing a protective role by inhibiting intimal hyperplasia (Figure 12). This study explored the mechanism by which HTJDTLD prevents ISR after PCI from the perspective of proliferation, ERS, and autophagy and provides a pharmacological basis for the clinical application of HTJDTLD.

## Figures and Tables

**Table 1:** The relative abundance of the six most abundant components in HTJDTLD (positive ion mode).

Name	Formula	Molecular weight	RT (min)	Molecular weight (theoretical value)	△ppm	Area	Relative abundance
Galactinol	C_12_H_22_O_11_	342.11603	0.782	342.11621	0	2715591258	98.52
DL-arginine	C_6_H_14_N_4_O_2_	174.11173	0.728	174.11168	0	2644881072	96.15
Choline	C_5_H_13_NO	103.1001	0.748	103.09971	3	1725458496	65.39
D-(+)-proline	C_5_H_9_NO_2_	115.06358	0.799	115.06333	2	1696509265	63.47
Senkyunolide A	C_12_H_16_O_2_	192.11507	8.658	192.11503	0	1177889526	13.57
Chlorogenic acid	C_16_H_18_O_9_	354.0949	5.248	354.09508	0	1121635893	12.96

## Data Availability

The datasets used and/or analyzed during the current study are available from the corresponding author on reasonable request.

## Supplementary Materials

Supplementary Materials File S1: results of full-spectrum identification.

## References

[1] Virani S. S., Alonso A., Benjamin E. J. (2020). Heart disease and stroke statistics-2020 update: a report from the American heart association. *Circulation*.

[2] Levine G. N., Bates E. R., Blankenship J. C. (2016). 2015 ACC/AHA/SCAI focused update on primary percutaneous coronary intervention for patients with ST-elevation myocardial infarction: an update of the 2011 ACCF/AHA/SCAI guideline for percutaneous coronary intervention and the 2013 ACCF/AHA guideline for the management of ST-elevation myocardial infarction: a report of the American college of cardiology/American heart association task force on clinical practice guidelines and the society for cardiovascular angiography and interventions. *Circulation*.

[3] Iqbal J., Gunn J., Serruys P. W. (2013). Coronary stents: historical development, current status and future directions. *British Medical Bulletin*.

[4] Byrne R. A., Joner M., Kastrati A. (2015). Stent thrombosis and restenosis: what have we learned and where are we going? the andreas grüntzig lecture ESC 2014. *European Heart Journal*.

[5] Otsuka F., Byrne R. A., Yahagi K. (2015). Neoatherosclerosis: overview of histopathologic findings and implications for intravascular imaging assessment. *European Heart Journal*.

[6] Hamed S., Roguin A. (2006). [Endothelial progenitor cells and atherosclerosis]. *Harefuah*.

[7] Yang S., Wu M., Li X. (2020). Role of endoplasmic reticulum stress in atherosclerosis and its potential as a therapeutic target. *Oxidative Medicine and Cellular Longevity*.

[8] Schröder M. (2008). Endoplasmic reticulum stress responses. *Cellular and Molecular Life Sciences*.

[9] Kim C., Kim B. (2018). Anti-cancer natural products and their bioactive compounds inducing ER stress-mediated apoptosis: a review. *Nutrients*.

[10] Kaufman R. J. (2002). Orchestrating the unfolded protein response in health and disease. *Journal of Clinical Investigation*.

[11] Ron D. (2002). Translational control in the endoplasmic reticulum stress response. *Journal of Clinical Investigation*.

[12] Moon H.-S., Kim B., Gwak H., Suh D. H., Song Y. S. (2016). Autophagy and protein kinase RNA-like endoplasmic reticulum kinase (PERK)/eukaryotic initiation factor 2 alpha kinase (eIF2*α*) pathway protect ovarian cancer cells from metformin-induced apoptosis. *Molecular Carcinogenesis*.

[13] Storniolo A., Alfano V., Carbotta S., Ferretti E., Di Renzo L. (2018). IRE1*α* deficiency promotes tumor cell death and eIF2*α* degradation through PERK dipendent autophagy. *Cell Death Discovery*.

[14] Liu C., Yan D. Y., Wang C. (2020). Manganese activates autophagy to alleviate endoplasmic reticulum stress-induced apoptosis via PERK pathway. *Journal of Cellular and Molecular Medicine*.

[15] Kouroku Y., Fujita E., Tanida I. (2007). ER stress (PERK/eIF2*α* phosphorylation) mediates the polyglutamine-induced LC3 conversion, an essential step for autophagy formation. *Cell Death & Differentiation*.

[16] Noda N. N., Fujioka Y., Hanada T., Ohsumi Y., Inagaki F. (2013). Structure of the Atg12-Atg5 conjugate reveals a platform for stimulating Atg8-PE conjugation. *EMBO Reports*.

[17] Plácido A. I., Pereira C. M. F., Duarte A. I. (2014). The role of endoplasmic reticulum in amyloid precursor protein processing and trafficking: implications for Alzheimer’s disease. *Biochimica et biophysica acta*.

[18] Liao X., Sluimer J. C., Wang Y. (2012). Macrophage autophagy plays a protective role in advanced atherosclerosis. *Cell Metabolism*.

[19] Perrotta I. (2013). The use of electron microscopy for the detection of autophagy in human atherosclerosis. *Micron*.

[20] Song S., Tan J., Miao Y., Li M., Zhang Q. (2017). Crosstalk of autophagy and apoptosis: involvement of the dual role of autophagy under ER stress. *Journal of Cellular Physiology*.

[21] Schmidt T., Abbott J. D. (2018). Coronary stents: history, design, and construction. *Journal of Clinical Medicine*.

[22] Al Suwaidi J., Berger P. B., Holmes D. R. (2000). Coronary artery stents. *Jama*.

[23] Björnsson E., Jacobsen E. I., Kalaitzakis E. (2012). Hepatotoxicity associated with statins: reports of idiosyncratic liver injury post-marketing. *Journal of Hepatology*.

[24] Demyen M., Alkhalloufi K., Pyrsopoulos N. T. (2013). Lipid-lowering agents and hepatotoxicity. *Clinics in Liver Disease*.

[25] Costa F., Van Klaveren D., Feres F. (2019). Dual antiplatelet therapy duration based on ischemic and bleeding risks after coronary stenting. *Journal of the American College of Cardiology*.

[26] Jacobs A. K., Nedeljkovic Z. S. (2016). Dual-antiplatelet therapy. *JACC: Cardiovascular Interventions*.

[27] Braghetto I., Csendes A., Lazo M., Smok G., Chamorro A. (1982). [Pancreatic cystadenoma. Report of 4 clinical cases]. *Revista medica de Chile*.

[28] Zou G., Zhu J., Liu Z. (2019). Detoxification and activating blood circulation decoction reduces restenosis involving the TLR4/NF-*κ*B pathway after balloon injury. *Prostaglandins & Other Lipid Mediators*.

[29] Wu L., Zhang W., Li H. (2009). Inhibition of aortic intimal hyperplasia and cell cycle protein and extracellular matrix protein expressions by BuYang HuanWu decoction. *Journal of Ethnopharmacology*.

[30] Petrasheskaya N., Tae H.-J., Ahmet I. (2016). A rat carotid balloon injury model to test anti-vascular remodeling therapeutics. *Journal of Visualized Experiments: JoVE*.

[31] Yang H., Xue Y., Kuang S. (2019). Involvement of Orai1 in tunicamycin-induced endothelial dysfunction. *The Korean Journal of Physiology & Pharmacology*.

[32] Takaguri A., Kubo T., Mori M., Satoh K. (2017). The protective role of YAP1 on ER stress-induced cell death in vascular smooth muscle cells. *European Journal of Pharmacology*.

[33] Dang H., Song B., Dong R., Zhang H. (2018). Atorvastatin reverses the dysfunction of human umbilical vein endothelial cells induced by angiotensin II. *Experimental and Therapeutic Medicine*.

[34] Sriram V., Patterson C. (2001). Cell cycle in vasculoproliferative diseases. *Circulation*.

[35] Park S.-J., Kang S.-J., Virmani R., Nakano M., Ueda Y. (2012). In-stent neoatherosclerosis. *Journal of the American College of Cardiology*.

[36] Gao Y., Gao C.-Y., Zhu P. (2018). Ginsenoside Re inhibits vascular neointimal hyperplasia in balloon-injured carotid arteries through activating the eNOS/NO/cGMP pathway in rats. *Biomedicine & Pharmacotherapy*.

[37] Kelman Z. (1997). PCNA: structure, functions and interactions. *Oncogene*.

[38] Miano J. M., Berk B. C. (2000). Retinoids. *Circulation Research*.

[39] Xue C.-D., Chen Y., Ren J.-L. (2019). Endogenous intermedin protects against intimal hyperplasia by inhibiting endoplasmic reticulum stress. *Peptides*.

[40] Hong J., Kim K., Kim J.-H., Park Y. (2017). The role of endoplasmic reticulum stress in cardiovascular disease and exercise. *International Journal of Vascular Medicine*.

[41] Ron D., Walter P. (2007). Signal integration in the endoplasmic reticulum unfolded protein response. *Nature Reviews Molecular Cell Biology*.

[42] Jin J.-K., Blackwood E. A., Azizi K. (2017). ATF6 decreases myocardial ischemia/reperfusion damage and links ER stress and oxidative stress signaling pathways in the heart. *Circulation Research*.

[43] Zeng L., Li Y., Yang J. (2015). XBP 1-deficiency abrogates neointimal lesion of injured vessels via cross talk with the PDGF signaling. *Arteriosclerosis, Thrombosis, and Vascular Biology*.

[44] Wang B., Zhang M., Urabe G. (2020). PERK inhibition mitigates restenosis and thrombosis. *JACC: Basic to Translational Science*.

[45] Yao S., Tian H., Miao C. (2015). D4F alleviates macrophage-derived foam cell apoptosis by inhibiting CD36 expression and ER stress-CHOP pathway. *Journal of Lipid Research*.

[46] Tabas I. (2010). The role of endoplasmic reticulum stress in the progression of atherosclerosis. *Circulation Research*.

[47] Høyer-Hansen M., Jäättelä M. (2007). Connecting endoplasmic reticulum stress to autophagy by unfolded protein response and calcium. *Cell Death and Differentiation*.

[48] Yorimitsu T., Nair U., Yang Z., Klionsky D. J. (2006). Endoplasmic reticulum stress triggers autophagy. *Journal of Biological Chemistry*.

[49] Wang J., Kang R., Huang H. (2014). Hepatitis C virus core protein activates autophagy through EIF2AK3 and ATF6 UPR pathway-mediated MAP1LC3B and ATG12 expression. *Autophagy*.

[50] Zhou B., Lu Q., Liu J. (2019). Melatonin increases the sensitivity of hepatocellular carcinoma to sorafenib through the PERK-ATF4-beclin1 pathway. *International Journal of Biological Sciences*.

[51] Okopień B., Bułdak Ł, Bołdys A. (2016). Current and future trends in the lipid lowering therapy. *Pharmacological Reports: PR*.

[52] Yamawaki T., Yamada A., Fukumoto Y. (2007). Statin therapy may prevent restenosis after successful coronary intervention, independent of lipid-lowering effect and CRP level. *Fukuoka Igaku Zasshi*.

[53] Prasad K. (2013). Do statins have a role in reduction/prevention of post-PCI restenosis?. *Cardiovascular Therapeutics*.

[54] Guo X., Wang L., Xia X., Wang P., Li X. (2019). Effects of atorvastatin and/or probucol on recovery of atherosclerosis in high-fat-diet-fed apolipoprotein E-deficient mice. *Biomedicine & Pharmacotherapy*.

[55] Zhu B., Gong Y., Yan G. (2017). Atorvastatin treatment modulates p16 promoter methylation to regulate p16 expression. *The FEBS Journal*.

[56] Xiong W., Fei M., Wu C. (2020). Atorvastatin inhibits endoplasmic reticulum stress through AMPK signaling pathway in atherosclerosis in mice. *Experimental and Therapeutic Medicine*.

[57] Carrascosa M. F., Salcines-Caviedes J. R., Lucena M. I., Andrade R. J. (2015). Acute liver failure following atorvastatin dose escalation: is there a threshold dose for idiosyncratic hepatotoxicity?. *Journal of Hepatology*.

[58] Li S., Yu Y., Jin Z. (2019). Prediction of pharmacokinetic drug-drug interactions causing atorvastatin-induced rhabdomyolysis using physiologically based pharmacokinetic modelling. *Biomedicine & Pharmacotherapy*.

[59] Xu C., Bailly-Maitre B., Reed J. C. (2005). Endoplasmic reticulum stress: cell life and death decisions. *Journal of Clinical Investigation*.

[60] Ishimura S., Furuhashi M., Mita T. (2014). Reduction of endoplasmic reticulum stress inhibits neointima formation after vascular injury. *Scientific Reports*.

[61] Wang W., Zhang Y., Hui H. (2021). The effect of endothelial progenitor cell transplantation on neointimal hyperplasia and reendothelialisation after balloon catheter injury in rat carotid arteries. *Stem Cell Research & Therapy*.

