# Supplementary material for: Huotan Jiedu Tongluo Decoction Inhibits Balloon-Injury-Induced Carotid Artery Intimal Hyperplasia in the Rat through the PERK-eIF2α-ATF4 Pathway and Autophagy Mediation
Source: Evid Based Complement Alternat Med. 2021 Jul 20;2021:5536237. doi: 10.1155/2021/5536237 (PMC8318774; doi:10.1155/2021/5536237)
Supplement: Supplementary Materials — File S1: results of full-spectrum identification. [file 5536237.f1.pdf]

Positive ion mode

Name	Formula	CAS_num	Class	Molecular Weight	RT [min]	Molecular Weight(Theoretical value)	△ppm	Area: zhongyaofufangdongganfen-P.raw (F2)	Relative concentration of each substance in HTJDTLD (ug/ml)
Salvianolic acid B	C36 H30 O16	121521-90-2	2-arylbenzofuran flavonoids	718.15345	7.782	718.15338	0	103087960.9	4.913
Lithospermic acid	C27 H22 O12	28831-65-4	2-arylbenzofuran flavonoids	538.11144	7.774	538.11113	0	358070403.2	17.065
1-[2-(1,3-Benzodioxol-5-yl)-3-methyl-1-benzofuran-5-yl]-1,2-propanediol	C19 H18 O5	NA	2-arylbenzofuran flavonoids	326.11529	11.657	326.11542	0	23196873.73	1.106
N-(2-AMINOETHYL)ETHYLENEUREA	C5 H11 N3 O	6281-42-1	Azolidines	129.09037	0.839	129.09021	1	247118583.8	11.777
4-Ethynylaniline	C8 H7 N	14235-81-5	Benzene and substituted derivatives	117.05808	4.518	117.05785	1	45798517.73	2.183
2,4-Dimethylbenzaldehyde	C9 H10 O	15764-16-6	Benzene and substituted derivatives	134.07324	9.393	134.07316	0	30600048.8	1.458
3',4'-Dihydroxyphenylacetone	C9 H10 O3	2503-44-8	Benzene and substituted derivatives	166.06312	8.494	166.06299	0	125432247.9	5.978
3',4'-Dihydroxyphenylacetone	C9 H10 O3	2503-44-8	Benzene and substituted derivatives	166.06311	2.958	166.06299	0	111246208.4	5.302
4-Phenylcyclohexanone	C12 H14 O	4894-75-1	Benzene and substituted derivatives	174.1045	8.657	174.10447	0	211949771.6	10.101
Phenylglyoxylic acid	C8 H6 O3	611-73-4	Benzene and substituted derivatives	150.03179	5.358	150.03169	0	587510140.2	28.000
Phenacetin	C10 H13 N O2	62-44-2	Benzene and substituted derivatives	179.09474	1.115	179.09463	0	42302224.75	2.016
Phthaldialdehyde	C8 H6 O2	643-79-8	Benzene and substituted derivatives	134.03691	5.249	134.03678	0	88914901.45	4.238
4,7-dihydroxymellein_130075	C10 H10 O5	NA	Benzopyrans	210.05284	5.223	210.05282	0	142304211.4	6.782
Boc-Asp-OH	C9 H15 N O6	13726-67-5	Carboxylic acids and derivatives	233.08985	0.774	233.08994	0	683543664.1	32.576
D-(+)-Pipecolinic acid	C6 H11 N O2	1723-00-8	Carboxylic acids and derivatives	129.07918	0.847	129.07898	1	248008034.9	11.820
D-(+)-Proline	C5 H9 N O2	344-25-2	Carboxylic acids and derivatives	115.06358	0.799	115.06333	2	1696509265	80.853
DL-Stachydrine	C7 H13 N O2	4136-37-2	Carboxylic acids and derivatives	143.09473	0.828	143.09463	0	236314220.6	11.262
L-Glutamic acid	C5 H9 N O4	56-86-0	Carboxylic acids and derivatives	147.05321	0.78	147.05316	0	360846116.9	17.197
Metalaxyl	C15 H21 N O4	57837-19-1	Carboxylic acids and derivatives	279.14699	4.697	279.14706	0	13875073.59	0.661
DL-Glutamine	C5 H10 N2 O3	585-21-7	Carboxylic acids and derivatives	146.0692	0.769	146.06914	0	212821887.9	10.143
L-Phenylalanine	C9 H11 N O2	63-91-2	Carboxylic acids and derivatives	165.07913	2.249	165.07898	0	211273475.8	10.069
Latamoxef	C20 H20 N6 O9 S	64952-97-2	Carboxylic acids and derivatives	520.101	7.776	520.10125	0	779731307	37.161
DL-Arginine	C6 H14 N4 O2	7200-25-1	Carboxylic acids and derivatives	174.11173	0.728	174.11168	0	2644881072	126.050
L-Valine	C5 H11 N O2	72-18-4	Carboxylic acids and derivatives	117.07923	0.799	117.07898	2	262766638.6	12.523
L-Isoleucine	C6 H13 N O2	73-32-5	Carboxylic acids and derivatives	131.0948	1.323	131.09463	1	425301786.7	20.269
L-Pyroglutamic acid	C5 H7 N O3	98-79-3	Carboxylic acids and derivatives	129.04276	1.139	129.04259	1	721064320.8	34.365
N-(2,6-Dimethylphenyl)-N-(methoxyacetyl) alanine	C14 H19 N O4	NA	Carboxylic acids and derivatives	265.13155	1.225	265.13141	0	35268754.43	1.681
N-Fructosyl isoleucine	C12 H23 N O7	NA	Carboxylic acids and derivatives	293.14737	1.318	293.14745	0	505362969.2	24.085
N-Fructosyl phenylalanine	C15 H21 N O7	NA	Carboxylic acids and derivatives	327.13172	2.152	327.1318	0	110231589.1	5.253
Caffeic acid	C9 H8 O4	331-39-5	Cinnamic acids and derivatives	180.04231	4.47	180.04226	0	159683453.5	7.610
Caffeic acid	C9 H8 O4	331-39-5	Cinnamic acids and derivatives	180.04232	7.466	180.04226	0	138959515.2	6.623
Caffeic acid	C9 H8 O4	331-39-5	Cinnamic acids and derivatives	180.04231	7.265	180.04226	0	85475345.96	4.074
Sinapic acid	C11 H12 O5	530-59-6	Cinnamic acids and derivatives	224.06847	6.086	224.06847	0	92489552.55	4.408
Isoferulic acid	C10 H10 O4	537-73-5	Cinnamic acids and derivatives	194.05801	4.774	194.05791	0	24318380.73	1.159
Ferulic acid	C10 H10 O4	537-98-4	Cinnamic acids and derivatives	194.05802	6.888	194.05791	0	55734122.98	2.656
2-Hydroxycinnamic acid	C9 H8 O3	614-60-8	Cinnamic acids and derivatives	164.04753	1.226	164.04734	1	98369375.31	4.688
4-Methoxycinnamic acid	C10 H10 O3	830-09-1	Cinnamic acids and derivatives	178.06319	8.015	178.06299	1	13031352.34	0.621
Aflatoxin B1	C17 H12 O6	1162-65-8	Coumarins and derivatives	312.06319	7.775	312.06339	0	23778699.57	1.133
4-Methylumbelliferyl-α-D-glucopyranoside	C16 H18 O8	17833-43-1	Coumarins and derivatives	338.10015	7.088	338.10017	0	65866231.79	3.139
4-Methylumbelliferone	C10 H8 O3	90-33-5	Coumarins and derivatives	176.04744	5.361	176.04734	0	88571979.41	4.221
7-Hydroxycoumarine	C9 H6 O3	93-35-6	Coumarins and derivatives	162.03174	5.248	162.03169	0	924490140.7	44.060
Uracil	C4 H4 N2 O2	66-22-8	Diazines	112.02765	1.147	112.02728	3	21261639	1.013
Cytosine	C4 H5 N3 O	71-30-7	Diazines	111.04359	0.821	111.04326	2	36861177.56	1.757
Sarracenin	C11 H14 O5	59653-37-1	Dioxanes	226.08412	6.342	226.08412	0	276811035.6	13.192
9-Oxo-ODE	C18 H30 O3	NA	Fatty Acyls	294.21932	9.878	294.21949	0	21036162.67	1.003
9S,13R-12-Oxophytodienoic acid	C18 H28 O3	NA	Fatty Acyls	292.20368	9.527	292.20384	0	27576581.21	1.314
S(8)-(2-methylpropanoyl)dihydrolipoamide	C12 H23 N O2 S2	NA	Fatty Acyls	277.11601	0.815	277.11702	-3	219804423.4	10.475
Quercetin	C15 H10 O7	117-39-5	Flavonoids	302.0425	6.684	302.04265	0	17934549.01	0.855
Rutin	C27 H30 O16	153-18-4	Flavonoids	610.15386	7.515	610.15338	0	23448530.16	1.118
Keracyanin	C27 H30 O15	28338-59-2	Flavonoids	594.15898	6.587	594.15847	0	16652244.37	0.794
Neodiosmin	C28 H32 O15	38665-01-9	Flavonoids	608.17467	7.043	608.17412	0	15658630.8	0.746
Quercetin-3β-D-glucoside	C21 H20 O12	482-35-9	Flavonoids	464.09556	6.686	464.09548	0	51642967.48	2.461
Afzelin	C21 H20 O10	482-39-3	Flavonoids	432.10561	8.542	432.10565	0	11507316.28	0.548
Kaempferol	C15 H10 O6	520-18-3	Flavonoids	286.04744	9.853	286.04774	-1	31315176.6	1.492
Kaempferol	C15 H10 O6	520-18-3	Flavonoids	286.04744	7.559	286.04774	-1	12146583.69	0.579
Quercitrin	C21 H20 O11	522-12-3	Flavonoids	448.1006	7.822	448.10056	0	57394027.33	2.735
Cynaroside	C21 H20 O11	5373-11-5	Flavonoids	448.1006	6.689	448.10056	0	56319762.41	2.684
4',7-Dihydroxyflavanone	C15 H12 O4	69097-97-8	Flavonoids	256.07347	6.661	256.07356	0	281721656.3	13.426
4',7-Dihydroxyflavanone	C15 H12 O4	69097-97-8	Flavonoids	256.07347	8.739	256.07356	0	27567568.94	1.314
Cantharidin	C10 H12 O4	56-25-7	Furofurans	196.07373	4.83	196.07356	0	51773441.7	2.467
Cantharidin	C10 H12 O4	56-25-7	Furofurans	196.07368	5.803	196.07356	0	257119310.3	12.254
2,3,4,9-Tetrahydro-1H-β-carboline-3-carboxylic acid	C12 H12 N2 O2	82789-18-2	Harmala alkaloids	216.08991	5.431	216.08988	0	25800808.9	1.230
Hypoxanthine	C5 H4 N4 O	68-94-0	Imidazopyrimidines	136.03864	1.116	136.03851	0	30109779.92	1.435
Adenine	C5 H5 N5	73-24-5	Imidazopyrimidines	135.0546	0.836	135.0545	0	187969881.4	8.958
Guanine	C5 H5 N5 O	73-40-5	Imidazopyrimidines	151.0495	0.845	151.04941	0	32413530.46	1.545
4-Indolecarbaldehyde	C9 H7 N O	1047-86-6	Indoles and derivatives	145.05278	4.55	145.05276	0	26993897.26	1.286
trans-3-Indoleacrylic acid	C11 H9 N O2	1204-06-4	Indoles and derivatives	187.06334	4.551	187.06333	0	344377146.4	16.412
Senkyunolide A	C12 H16 O2	63038-10-8	Isobenzofurans	192.11507	8.658	192.11503	0	1177889526	56.136
Formononetin	C16 H12 O4	485-72-3	Isoflavonoids	268.07335	10.435	268.07356	0	13823096.57	0.659
Ononin	C22 H22 O9	486-62-4	Isoflavonoids	430.12651	7.769	430.12638	0	75374284.8	3.592
6-Acetylmorphine	C19 H21 N O4	2784-73-8	Morphinans	327.14714	5.592	327.14706	0	17876664.46	0.852
3,5-Dihydroxy-2-naphthoic acid	C11 H8 O4	89-35-0	Naphthalenes	204.04244	7.492	204.04226	0	11313227.02	0.539
2-Naphthylamine	C10 H9 N	91-59-8	Naphthalenes	143.07358	5.431	143.0735	0	21445912.51	1.022
Diethylpyrocarbonate	C6 H10 O5	1609-47-8	Organic carbonic acids and derivatives	162.05282	0.798	162.05282	0	246906910.9	11.767
(2,3-Dihydroxypropyl)arsonic acid	C3 H9 As O5	NA	Organometalloid compounds	199.96574	0.67	199.96659	-4	296835570.3	14.147
Acetyl-β-methylcholine	C8 H17 N O2	55-92-5	Organonitrogen compounds	159.12602	0.839	159.12593	0	49633002.03	2.365
Choline	C5 H13 N O	62-49-7	Organonitrogen compounds	103.1001	0.748	103.09971	3	1725458496	82.232
Maltotriose	C18 H32 O16	1109-28-0	Organooxygen compounds	504.16898	0.771	504.16903	0	468079672	22.308
Muramic acid	C9 H17 N O7	1114-41-6	Organooxygen compounds	251.1004	0.775	251.1005	0	90822871.32	4.328
4-Hydroxybenzaldehyde	C7 H6 O2	123-08-0	Organooxygen compounds	122.03705	5.367	122.03678	2	24622943.85	1.173
3,4-Dihydroxybenzaldehyde	C7 H6 O3	139-85-5	Organooxygen compounds	138.03183	5.029	138.03169	1	15178607.2	0.723
Sweroside	C16 H22 O9	14215-86-2	Organooxygen compounds	358.12625	5.802	358.12638	0	240123645.1	11.444
4,5-Dicaffeoylquinic acid	C25 H24 O12	14534-61-3	Organooxygen compounds	516.1269	7.263	516.12678	0	301305635.6	14.360
Heptanophenone	C13 H18 O	1671-75-6	Organooxygen compounds	190.13585	10.82	190.13577	0	13377518.78	0.638
2,3-dihydroxy-1-(4-hydroxy-3-methoxyphenyl)-propan-1-one	C10 H12 O5	168293-10-5	Organooxygen compounds	212.06847	5.36	212.06847	0	562112543.9	26.789
Swertiamarin	C16 H22 O10	17388-39-5	Organooxygen compounds	374.1212	5.362	374.1213	0	315133290.6	15.019
Amygdalin	C20 H27 N O11	29883-15-6	Organooxygen compounds	457.15859	6.596	457.15841	0	21276704.64	1.014
Chlorogenic acid	C16 H18 O9	327-97-9	Organooxygen compounds	354.0949	5.248	354.09508	0	1121635893	53.455
galactinol	C12 H22 O11	3687-64-7	Organooxygen compounds	342.11603	0.782	342.11621	0	2715591258	129.420
4-(Trifluoroacetyl)benzoic acid	C9 H5 F3 O3	58808-59-6	Organooxygen compounds	218.01923	0.762	218.01908	0	213702077.8	10.185
Glucose 1-phosphate	C6 H13 O9 P	59-56-3	Organooxygen compounds	260.02962	0.808	260.02972	0	50605636.01	2.412
5-Hydroxymethyl-2-furaldehyde	C6 H6 O3	67-47-0	Organooxygen compounds	126.03185	0.804	126.03169	1	207091796.4	9.870
Acetophenone	C8 H8 O	98-86-2	Organooxygen compounds	120.05775	9.394	120.05751	1	74845725.9	3.567
Acetophenone	C8 H8 O	98-86-2	Organooxygen compounds	120.05775	4.701	120.05751	1	26316373.51	1.254
7,8-Bis(hydroxymethyl)-1,4a-dimethyl-3,4,4a,5,6,7-hexahydro-2H-benzo[7]annulen-2-one	C15 H22 O3	NA	Organooxygen compounds	250.15684	10.266	250.15689	0	21421340.03	1.021
7,8-Bis(hydroxymethyl)-1,4a-dimethyl-3,4,4a,5,6,7-hexahydro-2H-benzo[7]annulen-2-one	C15 H22 O3	NA	Organooxygen compounds	250.15688	5.924	250.15689	0	14454106.62	0.689
(2S,3R)-2-Amino-1,3-heptadecanediol	C17 H37 N O2	NA	Others	287.28225	10.474	287.28243	0	324644349.5	15.472
(3R,5R)-1,3,5-Trihydroxy-4-{[(2E)-3-(4-hydroxy-3-methoxyphenyl)-2-propenoyl]oxy}cyclohexanecarboxylic acid	C17 H20 O9	NA	Others	368.11068	6.151	368.11073	0	32045593.51	1.527
(4E)-8-Hydroxy-4-(1-hydroxy-2-propanylidene)-10-oxatricyclo[7.2.1.01,5]dodecane-8-carboxylic acid	C15 H22 O5	NA	Others	282.14668	9.231	282.14672	0	16922684.49	0.807
2-Phenyl-N,N-bis(2-pyridinylmethyl)-1,3-thiazole-4-carboxamide	C22 H18 N4 O S	NA	Others	386.1187	2.96	386.12013	-3	85942540.56	4.096
3-(2-{2-[(3-Bromophenyl)sulfanyl]ethoxy}-3-methoxy-5-[(2S,5S)-5-(3,4,5-trimethoxyphenyl)tetrahydro-2-furanyl]phenyl)-1-butyl-1-hydroxyurea	C33 H41 Br N2 O8 S	NA	Others	704.17772	0.744	704.1767	1	758696105.2	36.158
4-(Dimethylamino)-N-[(4R,7S,8aS)-4-{3-[(4-fluorobenzyl)amino]-3-oxopropyl}-1-oxooctahydropyrrolo[1,2-a]pyrazin-7-yl]benzamide	C26 H32 F N5 O3	NA	Others	481.24861	13.106	481.24892	0	16518437.1	0.787
Methyl (1S,7R)-1-(β-D-glucopyranosyloxy)-7-methyl-5-oxo-1,4a,5,6,7,7a-hexahydrocyclopenta[c]pyran-4-carboxylate	C17 H24 O10	NA	Others	388.13683	6.24	388.13695	0	86284269.61	4.112
N-(4-Morpholinobenzyl)-2-thiophenesulfonamide	C15 H18 N2 O3 S2	NA	Oxazinanes	338.07659	4.707	338.07588	2	39379062.95	1.877
Acetylvanillin	C10 H10 O4	881-68-5	Phenol esters	194.05802	5.36	194.05791	0	223055708.4	10.630
3,4-dihydroxyphenylacetic acid	C8 H8 O4	102-32-9	Phenols	168.04235	5.224	168.04226	0	418320956.5	19.936
Guaiacol	C7 H8 O2	1990-05-1	Phenols	124.0528	5.36	124.05243	2	366037621.7	17.445
{1-[(4-Fluorophenyl)sulfonyl]-3-piperidinyl}methanol	C12 H16 F N O3 S	NA	Piperidines	273.08483	0.847	273.08349	4	229196003.1	10.923
(-)-Caryophyllene oxide	C15 H24 O	1139-30-6	Prenol lipids	220.18272	9.42	220.18272	0	24015434.53	1.145
Curcumenol	C15 H22 O2	19431-84-6	Prenol lipids	234.16197	9.394	234.16198	0	230517287.3	10.986
Cryptotanshinone	C19 H20 O3	35825-57-1	Prenol lipids	296.1409	13.277	296.14124	-1	199189557.4	9.493
Nootkatone	C15 H22 O	4674-50-4	Prenol lipids	218.16709	8.326	218.16707	0	14879309.4	0.709
18-β-Glycyrrhetinic acid	C30 H46 O4	471-53-4	Prenol lipids	470.33929	10.048	470.33961	0	210055817.7	10.011
Tanshinone IIA	C19 H18 O3	568-72-9	Prenol lipids	294.12537	14.456	294.12559	0	100505725.1	4.790
Dihydrotanshinone I	C18 H14 O3	87205-99-0	Prenol lipids	278.094	13.712	278.09429	-1	50529942.89	2.408
(3aR,8R,8aR,9aR)-8-Hydroxy-8a-methyl-3,5-bis(methylene)decahydronaphtho[2,3-b]furan-2(3H)-one	C15 H20 O3	NA	Prenol lipids	248.14116	10.349	248.14124	0	11769277.37	0.561
(3aS,10aR,10bR)-6,10a-Dimethyl-3-methylene-3,3a,4,5,7,8,10a,10b-octahydrofuro[3',2':6,7]cyclohepta[1,2-b]pyran-2,9-dione	C15 H18 O4	1860947	Prenol lipids	262.12044	7.958	262.12051	0	21088768.89	1.005
2-[(1S,2S,4aR,8aS)-1-hydroxy-4a-methyl-8-methylidene-1,2,3,4,5,6,7,8a-octahydronaphthalen-2-yl]prop-2-enoic acid	C15 H22 O3	NA	Prenol lipids	250.15689	8.819	250.15689	0	255490319.5	12.176
Methyl 1-(hexopyranosyloxy)-4a-hydroxy-7-methyl-5-oxo-1,4a,5,6,7,7a-hexahydrocyclopenta[c]pyran-4-carboxylate	C17 H24 O11	NA	Prenol lipids	404.13188	6.087	404.13186	0	99847936.62	4.759
Crotonoside	C10 H13 N5 O5	1818-71-9	Purine nucleosides	283.09157	1.321	283.09167	0	90678592.9	4.322
Adenosine	C10 H13 N5 O4	58-61-7	Purine nucleosides	267.09679	1.209	267.09675	0	306133715.2	14.590
4-Hydroxy-6-methyl-2-pyrone	C6 H6 O3	675-10-5	Pyrans	126.03183	5.802	126.03169	1	41831061.07	1.994
Methyl isonicotinate	C7 H7 N O2	2459-09-8	Pyridines and derivatives	137.04769	0.797	137.04768	0	214877117.8	10.241
Nicotinic acid	C6 H5 N O2	59-67-6	Pyridines and derivatives	123.03228	0.939	123.03203	2	13733804.86	0.655
Nicotinamide	C6 H6 N2 O	98-92-0	Pyridines and derivatives	122.04828	1.115	122.04801	2	26460550.49	1.261
19-Nortestosterone	C18 H26 O2	434-22-0	Steroids and steroid derivatives	274.1931	12.013	274.19328	0	11409521.25	0.544
Salvianolic acid A	C26 H22 O10	96574-01-5	Stilbenes	494.12123	7.776	494.1213	0	18761568.61	0.894
2-AMINO-3-(2-CHLORO-PHENYL)-PROPIONIC ACID	C9 H10 Cl NO2	14091-11-3	Internal standard	199.04004	4.503	199.04001	0	419655064	1.000

Negative ion mode

Name	Formula	CAS_num	Class	Molecular Weight	RT [min]	Molecular Weight(Theoretical value)	△ppm	Area: zhongyaofufangdongganfen-N.raw (F2)	Relative concentration of each substance in HTJDTLD (ug/ml)
Lithospermic acid	C27 H22 O12	28831-65-4	2-arylbenzofuran flavonoids	538.11122	7.498	538.11113	0	126174105.7	61.782
4-[(E)-3-[1-carboxy-2-(3,4-dihydroxyphenyl)ethoxy]-3-oxoprop-1-enyl]-2-(3,4-dihydroxyphenyl)-7-hydroxy-2,3-dihydro-1-benzofuran-3-carboxylic acid	C27 H22 O12	NA	2-arylbenzofuran flavonoids	538.11122	7.11	538.11113	0	123990630.6	60.713
(2R)-2-[(E)-3-[3-[(1R)-1-carboxy-2-(3,4-dihydroxyphenyl)ethoxy]carbonyl-2-(3,4-dihydroxyphenyl)-7-hydroxy-2,3-dihydro-1-benzofuran-4-yl]prop-2-enoyl]oxy-3-(3,4-dihydroxyphenyl)propanoic acid	C36 H30 O16	NA	2-arylbenzofuran flavonoids	718.15413	7.776	718.15338	1	4622354482	2263.382
Homogentisic acid	C8 H8 O4	451-13-8	Benzene and substituted derivatives	168.04147	2.935	168.04226	-4	8155324.273	3.993
Gallic acid	C7 H6 O5	149-91-7	Benzene and substituted derivatives	170.02075	1.764	170.02152	-4	480122967.5	235.097
Octyl gallate	C15 H22 O5	1034-01-1	Benzene and substituted derivatives	282.14683	9.228	282.14672	0	52053620.16	25.489
Picloxydine	C20 H24 Cl2 N10	5636-92-0	Benzene and substituted derivatives	474.15851	0.807	474.15625	4	2038381214	998.114
L-Phenylalanine	C9 H11 N O2	63-91-2	Carboxylic acids and derivatives	165.07817	2.204	165.07898	-4	23238723.53	11.379
cis-Aconitic acid	C6 H6 O6	585-84-2	Carboxylic acids and derivatives	174.01562	0.896	174.01644	-4	82067063.56	40.185
L-Tyrosine	C9 H11 N O3	60-18-4	Carboxylic acids and derivatives	181.07316	1.214	181.07389	-4	17683431.43	8.659
N-Fructosyl pyroglutamate	C11 H17 N O8	NA	Carboxylic acids and derivatives	291.09553	1.114	291.09542	0	230091024.6	112.666
beta-d-glucose pentaacetate	C16 H22 O11	4163-59-1	Carboxylic acids and derivatives	390.11639	5.215	390.11621	0	1780343757	871.763
beta-d-glucose pentaacetate	C16 H22 O11	4163-59-1	Carboxylic acids and derivatives	390.11639	2.586	390.11621	0	171363216.8	83.910
Caffeic acid	C9 H8 O4	331-39-5	Cinnamic acids and derivatives	180.0415	5.73	180.04226	-4	202576352.3	99.194
Ferulic acid	C10 H10 O4	537-98-4	Cinnamic acids and derivatives	194.05723	6.89	194.05791	-3	72987717.08	35.739
Hydroxyferulic acid	C10 H10 O5	1782-55-4	Cinnamic acids and derivatives	210.05225	5.082	210.05282	-2	89454388.09	43.802
Rosmarinic acid	C18 H16 O8	20283-92-5	Cinnamic acids and derivatives	360.08467	7.47	360.08452	0	495819855.3	242.783
[(Carbamoylamino)methyl]carbamate	C3 H6 N3 O3	NA	Diazines	132.04151	0.8	132.04092	4	1905636208	933.114
Suberic acid	C8 H14 O4	505-48-6	Fatty Acyls	174.08839	6.57	174.08921	-4	5993165.389	2.935
3-tert-Butyladipic acid	C10 H18 O4	10347-88-3	Fatty Acyls	202.11995	8.656	202.12051	-2	8800949.99	4.309
Oleic acid alkyne	C18 H30 O2	151333-45-8	Fatty Acyls	278.22444	15.895	278.22458	0	5426963.579	2.657
Corchorifatty acid F	C18 H32 O5	95341-44-9	Fatty Acyls	328.22514	9.528	328.22497	0	141261021	69.170
(15Z)-9,12,13-Trihydroxy-15-octadecenoic acid	C18 H34 O5	NA	Fatty Acyls	330.24077	9.883	330.24062	0	219651082.4	107.554
Luteolin	C15 H10 O6	491-70-3	Flavonoids	286.04774	9.853	286.04774	0	62617659.95	30.661
Catechin	C15 H14 O6	88191-48-4	Flavonoids	290.07893	5.884	290.07904	0	6661192.727	3.262
Quercetin	C15 H10 O7	117-39-5	Flavonoids	302.04265	8.972	302.04265	0	7057322.608	3.456
7-hydroxy-2-[4-[(2S,3R,4S,5S,6R)-3,4,5-trihydroxy-6-(hydroxymethyl)oxan-2-yl]oxyphenyl]-2,3-dihydrochromen-4-one	C21 H22 O9	NA	Flavonoids	418.12676	6.664	418.12638	0	503725979.9	246.654
Afzelin	C21 H20 O10	482-39-3	Flavonoids	432.10573	7.561	432.10565	0	36498324.1	17.872
7-Hydroxy-2-(4-hydroxyphenyl)-4-oxo-3,4-dihydro-2H-chromen-5-yl β-D-glucopyranoside	C21 H22 O10	NA	Flavonoids	434.12141	7.298	434.1213	0	20095955.59	9.840
Quercetin-3-O-rhamnoside	C21 H20 O11	522-12-3	Flavonoids	448.10077	7.825	448.10056	0	82466769.03	40.381
Kaempferol-7-O-glucoside	C21 H20 O11	16290-07-6	Flavonoids	448.10078	6.693	448.10056	0	121664360	59.574
Astragalin	C21 H20 O11	480-10-4	Flavonoids	448.10078	7.059	448.10056	0	23509946.38	11.512
Quercetin-3-O-rutinoside	C27 H30 O16	153-18-4	Flavonoids	610.15376	7.518	610.15338	0	70762143.43	34.649
Rutin	C27 H30 O16	153-18-4	Flavonoids	610.15401	6.46	610.15338	1	110562467.4	54.138
Viscumneoside V	C32 H40 O19	119016-92-1	Flavonoids	728.2173	5.356	728.21638	1	193837665.5	94.915
8-Cyclopentyltheophylline	C12 H16 N4 O2	35873-49-5	Imidazopyrimidines	248.12612	4.714	248.12733	-4	773459447.2	378.732
DL-Tryptophan	C11 H12 N2 O2	1954-12-6	Indoles and derivatives	204.08934	4.525	204.08988	-2	40727867.06	19.943
Formononetin	C16 H12 O4	485-72-3	Isoflavonoids	268.07353	8.716	268.07356	0	11321683.31	5.544
4-[4-(4-Hydroxy-3-methoxyphenyl)tetrahydro-1H,3H-furo[3,4-c]furan-1-yl]-2-methoxyphenyl hexopyranoside	C26 H32 O11	11042-30-1	Lignan glycosides	520.19417	6.921	520.19446	0	21520378.45	10.538
Lariciresinol 4-O-glucoside	C26 H34 O11	NA	Lignan glycosides	522.21026	6.526	522.21011	0	20609349.9	10.092
1,9b-Dihydroxy-6,6,9a-trimethyl-5,5a,6,7,8,9,9a,9b-octahydronaphtho[1,2-c]furan-3(1H)-one	C15 H22 O4	NA	Naphthofurans	266.15181	10.351	266.15181	0	43220221.42	21.163
Pseudouridine	C9 H12 N2 O6	1445-07-4	Nucleoside and nucleotide analogues	244.06937	1.145	244.06954	0	25519449.71	12.496
L-Iditol	C6 H14 O6	488-45-9	Organooxygen compounds	182.07823	0.751	182.07904	-4	115710254.2	56.659
D-(-)-Quinic acid	C7 H12 O6	77-95-2	Organooxygen compounds	192.06265	5.242	192.06339	-3	504206943.1	246.890
D-Saccharic acid	C6 H10 O8	87-73-0	Organooxygen compounds	210.03691	0.794	210.03757	-3	43260242.29	21.183
α,α-Trehalose	C12 H22 O11	99-20-7	Organooxygen compounds	342.1161	0.808	342.11621	0	5631515225	2757.527
Neochlorogenic acid	C16 H18 O9	906-33-2	Organooxygen compounds	354.09507	5.394	354.09508	0	1296457744	634.823
Chlorogenic acid	C16 H18 O9	327-97-9	Organooxygen compounds	354.09514	5.243	354.09508	0	3930805593	1924.758
Sweroside	C16 H22 O9	14215-86-2	Organooxygen compounds	358.12649	5.807	358.12638	0	998625431.5	488.987
3-O-Feruloylquinic acid	C17 H20 O9	62929-69-5	Organooxygen compounds	368.11076	6.127	368.11073	0	74295211.98	36.379
metamifop	C23 H18 Cl F N2 O4	256412-89-2	Organooxygen compounds	440.09266	0.79	440.09391	-2	944872245.4	462.666
Bioside	C20 H30 O12	NA	Organooxygen compounds	462.17406	4.482	462.17373	0	9161931.886	4.486
4,5-Dicaffeoylquinic acid	C25 H24 O12	14534-61-3	Organooxygen compounds	516.12702	7.267	516.12678	0	1597889674	782.423
STACHYOSE	C24 H42 O21	470-55-3	Organooxygen compounds	666.22242	0.748	666.22186	0	3068232998	1502.391
Angoroside C	C36 H48 O19	115909-22-3	Organooxygen compounds	784.2801	7.091	784.27898	1	214866462.1	105.211
4-[(Trifluoromethyl)sulfanyl]phenyl dihydrogen phosphate	C7 H6 F3 O4 P S	NA	Others	273.96629	0.675	273.96765	-4	295703579.2	144.794
1-(β-D-Glucopyranosyloxy)-7-methyl-1,4a,5,6,7,7a-hexahydrocyclopenta[c]pyran-4-carboxylic acid	C16 H24 O9	NA	Others	360.14206	6.386	360.14203	0	273312708.4	133.830
MFCD01051705	C17 H19 Cl N4 O2 S	NA	Others	378.09282	0.782	378.09172	2	1928508368	944.313
1-(3-Ethoxyphenyl)-7-fluoro-2-(4-methyl-2-pyridinyl)-1,2-dihydrochromeno[2,3-c]pyrrole-3,9-dione	C25 H19 F N2 O4	NA	Others	430.13241	0.859	430.13289	-1	227012094.3	111.159
N-(4-Sulfamoylphenyl)-2-(3,4,5-trimethoxybenzoyl)hydrazinecarbothioamide	C17 H20 N4 O6 S2	NA	Others	440.08409	0.858	440.08243	3	1665144987	815.355
2-[3,8-Dihydroxy-8-(hydroxymethyl)-3-methyl-2-oxodecahydro-5-azulenyl]-2-propanyl hexopyranoside	C21 H36 O10	NA	Others	448.23092	8.536	448.23085	0	246340008.3	120.623
Methyl 2-{[2-O-(6-deoxy-α-L-mannopyranosyl)-β-D-glucopyranosyl]oxy}benzoate	C20 H28 O12	NA	Others	460.15825	6.298	460.15808	0	8743955.912	4.282
1,6-Bis-O-(3,4,5-trihydroxybenzoyl)hexopyranose	C20 H20 O14	NA	Others	484.08525	5.079	484.08531	0	8934441.88	4.375
4,5-Di(4-biphenylyl)-2-(3,4-dimethoxyphenyl)-1H-imidazole	C35 H28 N2 O2	NA	Others	508.21589	6.804	508.21508	1	317510235.1	155.472
2-[(2Z)-3-(2-Furylmethyl)-2-{(2E)-[(2E)-2-methyl-3-phenyl-2-propen-1-ylidene]hydrazono}-4-oxo-1,3-thiazolidin-5-yl]-N-[3-(trifluoromethyl)phenyl]acetamide	C27 H23 F3 N4 O3 S	NA	Others	540.1457	0.77	540.1443	2	188676530.4	92.387
Liguiritigenin-7-O-β-D-apiosyl-4'-O-β-D-glucoside	C26 H30 O13	NA	Others	550.16954	7.436	550.16864	1	66150902.62	32.391
2-(3,4-Dihydroxyphenyl)-5,7-dihydroxy-4-oxo-4H-chromen-3-yl 6-O-β-D-xylopyranosyl-β-D-glucopyranoside	C26 H28 O16	NA	Others	596.1382	6.262	596.13773	0	14983736.38	7.337
2-(3,4-Dihydroxyphenyl)ethyl 3-O-(6-deoxy-β-L-mannopyranosyl)-6-O-[(2E)-3-(3,4-dihydroxyphenyl)-2-propenoyl]-β-D-glucopyranoside	C29 H36 O15	NA	Others	624.2057	6.612	624.20542	0	40775218.88	19.966
(3aS,6R,6aS,9aR,10aS,10bR)-2,8-Bis(4-benzoylphenyl)-6-[5-(hydroxymethyl)-2-furyl]-3a,4,6,6a,9a,10,10a,10b-octahydroisoindolo[5,6-e]isoindole-1,3,7,9(2H,8H)-tetrone	C45 H34 N2 O8	NA	Others	730.2328	6.159	730.23152	1	259747211.4	127.188
N-{4-[(4R,6S)-4-[4-(Hydroxymethyl)phenyl]-6-{[(5-methyl-1,3,4-thiadiazol-2-yl)sulfanyl]methyl}-1,3-dioxan-2-yl]benzyl}-Nalpha-[(4-methylphenyl)sulfonyl]-D-phenylalaninamide	C38 H40 N4 O6 S3	NA	Others	744.21223	5.227	744.211	1	557744277.3	273.105
(5Z,5'Z)-3,3'-(4-Benzoyl-1,2-phenylene)bis[5-(3-nitrobenzylidene)-2-phenyl-3,5-dihydro-4H-imidazol-4-one]	C45 H28 N6 O7	NA	Others	764.19978	0.761	764.20195	-2	339166878.1	166.076
N-(tert-Butoxycarbonyl)-L-tryptophyl-N~6~-[(2-methylphenyl)carbamoyl]-L-lysyl-L-alpha-aspartyl-Nalpha-methyl-L-phenylalaninamide	C44 H56 N8 O9	NA	Others	840.41536	10.344	840.41703	-1	273049989.6	133.702
N-(3-Chloro-4-morpholinophenyl)-6-oxo-1,4,5,6-tetrahydro-3-pyridazinecarboxamide	C15 H17 Cl N4 O3	NA	Oxazinanes	336.09776	4.682	336.09892	-3	48869722.7	23.930
3,4-Dihydroxyphenylacetic acid	C8 H8 O4	102-32-9	Phenols	168.04144	5.631	168.04226	-4	45124510.42	22.096
Danshensu	C9 H10 O5	76822-21-4	Phenylpropanoic acids	198.05229	3.009	198.05282	-2	115916540.4	56.760
Nardosinone	C15 H22 O3	23720-80-1	Prenol lipids	250.15678	8.819	250.15689	0	69549492.25	34.056
(±)-Abscisic acid	C15 H20 O4	14375-45-2	Prenol lipids	264.13603	8.549	264.13616	0	11448996.17	5.606
3-[(3aS,8R,8aR)-8-hydroxy-6,8-dimethyl-3-methylidene-2-oxo-3a,4,5,8a-tetrahydrocyclohepta[b]furan-7-yl]propanoic acid	C15 H20 O5	NA	Prenol lipids	280.13103	7.956	280.13107	0	127906493.4	62.631
Harpagide	C15 H24 O10	6926-8-5	Prenol lipids	364.13719	2.832	364.13695	0	910939749.4	446.051
Geniposidic acid	C16 H22 O10	27741-01-1	Prenol lipids	374.12136	4.749	374.1213	0	100901330.8	49.407
geniposidic acid	C16 H22 O10	27741-01-1	Prenol lipids	374.12143	5.355	374.1213	0	4979177776	2438.104
Mussaenosidic acid	C16 H24 O10	NA	Prenol lipids	376.13711	4.82	376.13695	0	351161141.1	171.950
Shanzhiside	C16 H24 O11	NA	Prenol lipids	392.13231	1.861	392.13186	1	92204999.27	45.149
Gardenoside	C17 H24 O11	NA	Prenol lipids	404.13186	5.429	404.13186	0	30448648.21	14.909
2-[3-ethenyl-5-methoxycarbonyl-2-[(2S,3R,4S,5S,6R)-3,4,5-trihydroxy-6-(hydroxymethyl)oxan-2-yl]oxy-3,4-dihydro-2H-pyran-4-yl]acetic acid	C17 H24 O11	NA	Prenol lipids	404.13193	6.088	404.13186	0	1175050311	575.375
Shanzhiside methyl ester	C17 H26 O11	NA	Prenol lipids	406.14767	5.055	406.14751	0	90457331.6	44.293
Lamiide	C17 H26 O12	27856-54-8	Prenol lipids	422.14293	4.312	422.14243	1	38213890.45	18.712
Harpagoside	C24 H30 O11	19210-12-9	Prenol lipids	494.1791	8.492	494.17881	0	522838910	256.013
[(2S,3R,4R)-3-ethenyl-5-methoxycarbonyl-2-[(2S,3R,4S,5S,6R)-3,4,5-trihydroxy-6-(hydroxymethyl)oxan-2-yl]oxy-3,4-dihydro-2H-pyran-4-yl]-4-oxobut-2-enyl]-2-[(2S,3R,4S,5S,6R)-3,4,5-trihydroxy-6-(hydroxymethyl)oxan-2-yl]o	C34 H46 O19	NA	Prenol lipids	758.26436	6.955	758.26333	1	244197165.5	119.574
Licoricesaponin G2	C42 H62 O17	118441-84-2	Prenol lipids	838.39992	9.768	838.3987	1	185006191.4	90.590
Cabotegravir	C19 H17 F2 N3 O5	1051375-10-0	Pyridines and derivatives	405.11171	0.801	405.11363	-4	384067931.1	188.063
Uridine monophosphate (UMP)	C9 H13 N2 O9 P	58-97-9	Pyrimidine nucleotides	324.0359	1.113	324.03587	0	14193804.07	6.950
Salvianolic acid A	C26 H22 O10	96574-01-5	Stilbenes	494.12147	7.497	494.1213	0	237090199.9	116.094
2-{[3-(Dimethylamino)propyl]amino}-3-(2-phenylethyl)-3,5,6,7,8,9-hexahydro-4H-cyclohepta[4,5]thieno[2,3-d]pyrimidin-4-one	C24 H32 N4 O S	NA	Thienopyrimidines	424.23086	8.534	424.22968	2	323834583.2	158.569
SR-9009	C20 H24 Cl N3 O4 S	1379686-30-2	Thiophenes	437.11687	5.354	437.1176	-1	241136449.7	118.075
FLUTRIMAZOLE	C22 H16 F2 N2	119006-77-8	Triphenyl compounds	346.12645	4.68	346.12815	-4	477489263.8	233.807
2-AMINO-3-(2-CHLORO-PHENYL)-PROPIONIC ACID	C9 H10 Cl NO2	14091-11-3	Internal standard	199.03937	4.475	199.04001	-3	40844675.16	1.000
